# Efficiency of acetate-based isopropanol synthesis in *Escherichia coli* W is controlled by ATP demand

**DOI:** 10.1186/s13068-024-02534-0

**Published:** 2024-08-05

**Authors:** Regina Kutscha, Tamara Tomin, Ruth Birner-Gruenberger, Pavlos Stephanos Bekiaris, Steffen Klamt, Stefan Pflügl

**Affiliations:** 1 Institute of Chemical, Environmental and Bioscience Engineering, Technische Universität Wien, Gumpendorfer Straße 1a, 1060 Vienna, Austria; 2 Christian Doppler Laboratory for Optimized Expression of Carbohydrate-Active Enzymes, Institute of Chemical, Environmental and Bioscience Engineering, Technische Universität Wien, Gumpendorfer Straße 1a, 1060 Vienna, Austria; 3 Institute of Chemical Technologies and Analytics, Technische Universität Wien, Vienna, Austria; 4 Analysis and Redesign of Biological Networks, Max Planck Institute for Dynamics of Complex Technical Systems, 39106 Magdeburg, Germany

**Keywords:** ATP demand, Metabolic modeling, Nitrogen starvation, Proteomics, Sustainable bioprocessing, Intracellular flux distribution

## Abstract

**Background:**

Due to increasing ecological concerns, microbial production of biochemicals from sustainable carbon sources like acetate is rapidly gaining importance. However, to successfully establish large-scale production scenarios, a solid understanding of metabolic driving forces is required to inform bioprocess design. To generate such knowledge, we constructed isopropanol-producing *Escherichia coli* W strains.

**Results:**

Based on strain screening and metabolic considerations, a 2-stage process was designed, incorporating a growth phase followed by a nitrogen-starvation phase. This process design yielded the highest isopropanol titers on acetate to date (13.3 g L^−1^). Additionally, we performed shotgun and acetylated proteomics, and identified several stress conditions in the bioreactor scenarios, such as acid stress and impaired sulfur uptake. Metabolic modeling allowed for an in-depth characterization of intracellular flux distributions, uncovering cellular demand for ATP and acetyl-CoA as limiting factors for routing carbon toward the isopropanol pathway. Moreover, we asserted the importance of a balance between fluxes of the NADPH-providing isocitrate dehydrogenase (ICDH) and the product pathway.

**Conclusions:**

Using the newly gained system-level understanding for isopropanol production from acetate, we assessed possible engineering approaches and propose process designs to maximize production. Collectively, our work contributes to the establishment and optimization of acetate-based bioproduction systems.

**Graphical Abstract:**

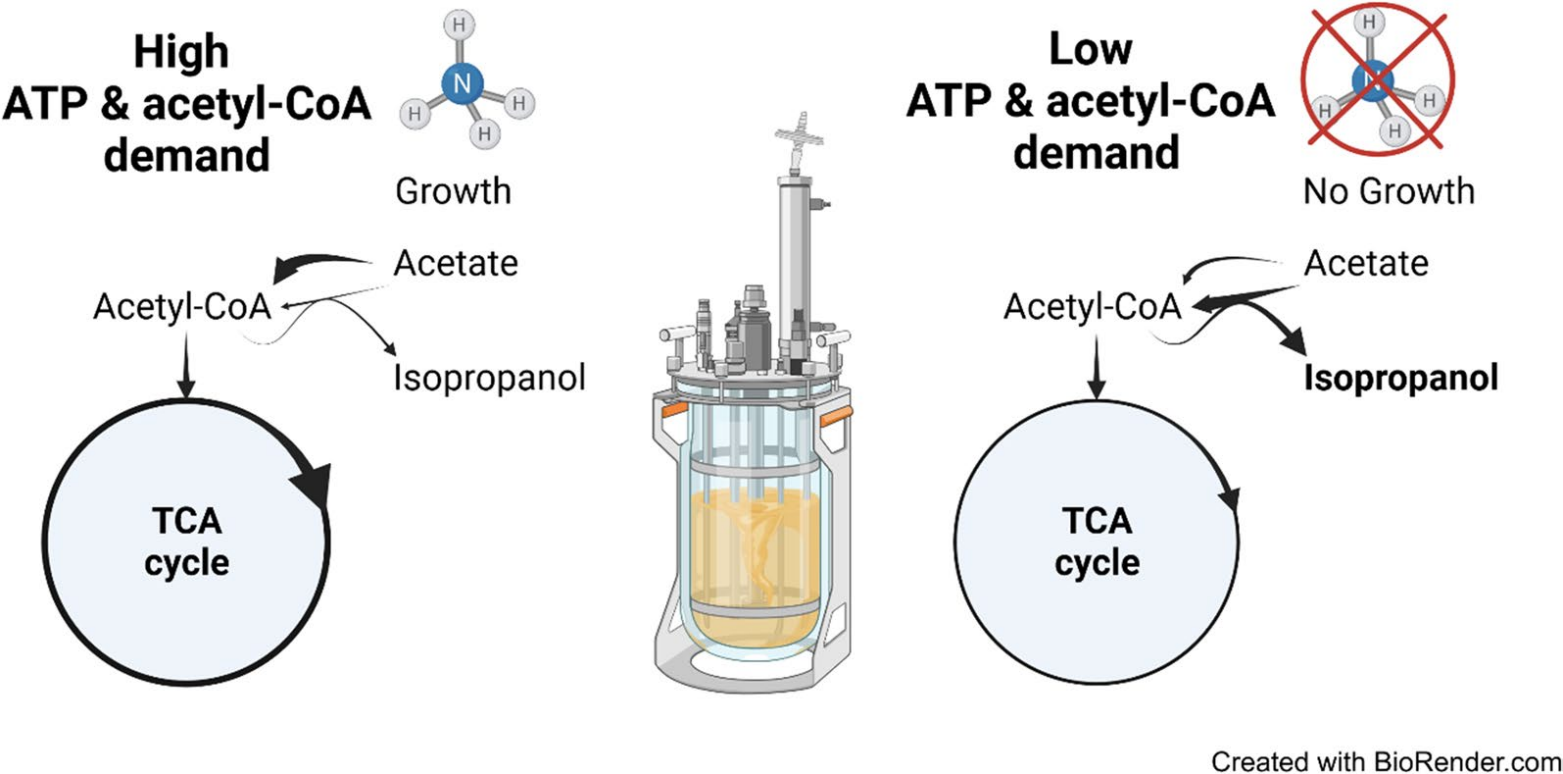

**Supplementary Information:**

The online version contains supplementary material available at 10.1186/s13068-024-02534-0.

## Background

With the arising need for ecological, microbial production of biochemicals and biofuels, considerable focus has been placed on utilizing sustainable carbon sources [1–6]. Such carbon sources should ideally be obtained cheaply to compete with fossil fuel-based production. Acetate, which can be readily derived from lignocellulosic and gaseous waste streams, fits these criteria perfectly [7–10]. Gas fermentation in particular enables a sustainable acetate supply [11, 12].

However, due to its toxicity at high concentrations and low energy content compared to sugars, its implementation into bioprocesses is somewhat challenging [13–15]. Energetic considerations are particularly important, because the ATP yield on acetate amounts to only 7 ATP, while substrates like glycerol and glucose support the formation of 15 and 26 ATP per molecule, respectively [16]. Nevertheless, in the past decade, multiple studies demonstrated the use of acetate for heterologous metabolite production, especially in *E. coli.* Obtained products include polyhydroxyalkanoates, naringenin, succinate, itaconic acid, isobutanol, and isopropanol [17–22]. Many of the pathways for these metabolites branch from the central carbon metabolism either from acetyl-CoA (polyhydroxyalkanoates, naringenin, isopropanol), the TCA cycle (succinate, itaconic acid, polyhydroxyalkanoates), or from pyruvate (isobutanol). The location of the branch node is important to consider for any pathway or strain optimization approach. For example, eliminating unwanted side product formation at the branch node can significantly increase production in the case of 2,3-butanediol [5]. Therefore, extensive metabolic characterization of any recombinant strains is necessary to further potential bioprocess development.

Isopropanol is a promising metabolite, because it has a wide range of applications as a solvent, a bulk chemical precursor, in cosmetics and in pharmaceutical products [23, 24]. While it is usually derived from fossil fuel-based sources, the need for a more sustainable production has also been recognized [24]. As of now, microbial processes do not yet have the capabilities to compete with conventional production. However, further research with sustainable carbon sources like acetate might eventually establish them as a viable alternative.

**Fig. 1:**
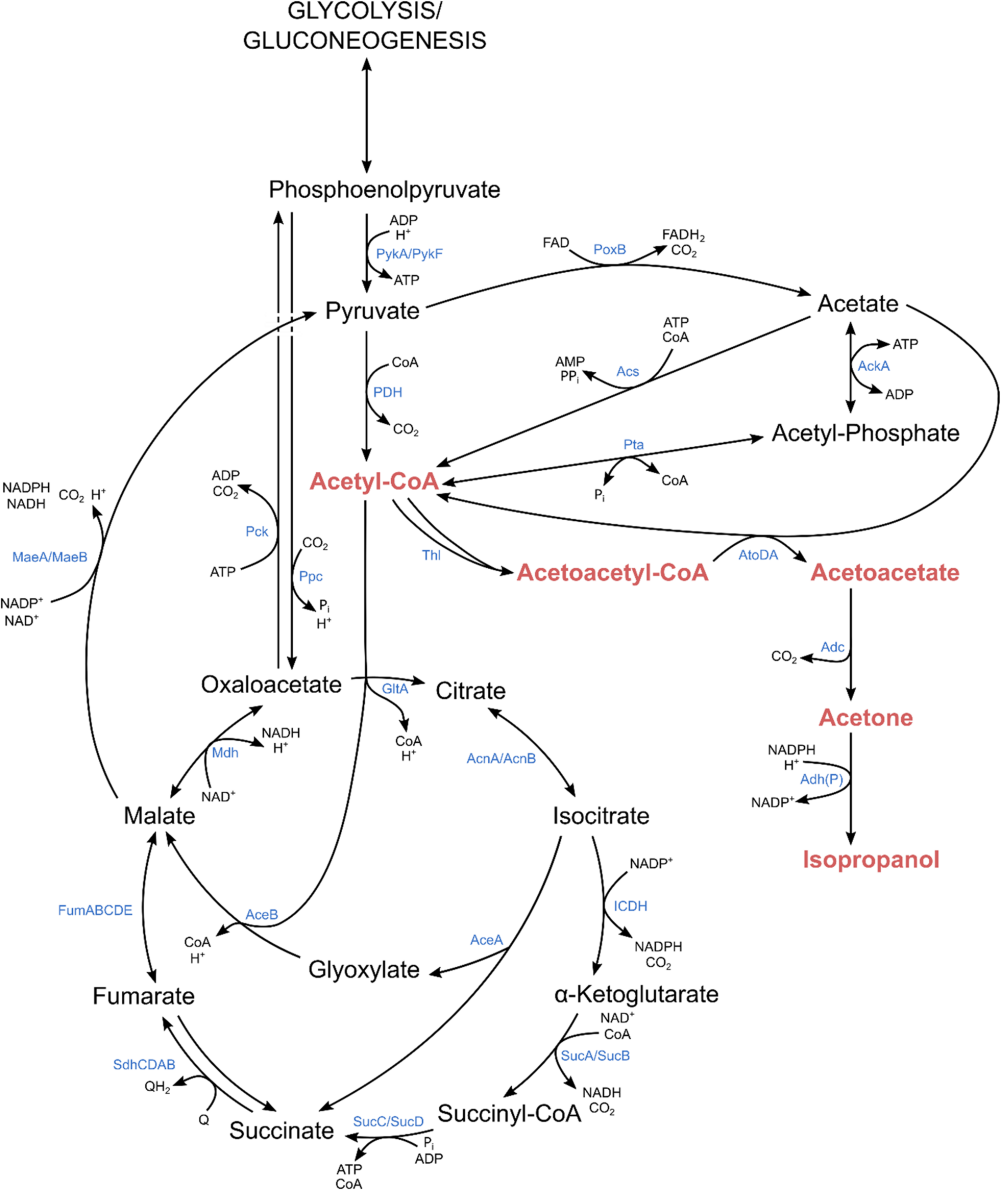
*E. coli* central carbon metabolism engineered for isopropanol production on acetate. *AckA* acetate kinase, *Pta* phosphate acetyltransferase, *Acs* acetyl-coenzyme A synthetase, *GltA* citrate synthase, *AcnA/AcnB* aconitate hydratase A/B, *ICDH* isocitrate dehydrogenase, *SucA* 2-oxoglutarate dehydrogenase E1 component, *SucB* dihydrolipoyllysine-residue succinyltransferase component of 2-oxoglutarate dehydrogenase complex, *SucC* succinate–CoA ligase [ADP-forming] subunit beta, *SucD* succinate-CoA ligase [ADP-forming] subunit alpha, *SdhCDAB* succinate dehydrogenase, *FumABCDE* fumarate hydratase, *Mdh* malate dehydrogenase, *AceA* isocitrate lyase, *AceB* malate synthase, *MaeA* NAD-dependent malic enzyme, *MaeB* NADP-dependent malic enzyme, *Pck* Phosphoenolpyruvate carboxykinase, Ppc phosphoenolpyruvate carboxylase, *PykA* pyruvate kinase II, *PykF* pyruvate kinase I, PDH pyruvate dehydrogenase, *PoxB* pyruvate dehydrogenase (ubiquinone), *Thl* thiolase, *AtoDA* acetate CoA-transferase, *Adc* acetoacetate decarboxylase, *Adh(P)* NADPH-dependent secondary alcohol dehydrogenase

To produce isopropanol in *E. coli*, a recombinant pathway extending from acetyl-CoA has to be introduced [25]. The pathway is depicted in Fig. 1.

Acetate is usually taken up via acetate kinase (AckA) and phosphate acetyltransferase (Pta) at high concentrations and converted to acetyl-CoA [26]. At this stage, the carbon either enters the TCA cycle (via citrate synthase, GltA) or is directed toward product formation (via thiolase, Thl). As carbon passes the acetate CoA-transferase (AtoDA), an additional acetate can be taken up “ATP-free”, thereby linking product formation and substrate uptake. The final reduction step from acetone to isopropanol requires NADPH as electron donor and is appropriated from the natural isopropanol producer *Clostridium beijerinckii*.

Currently, isopropanol production from acetate in *E. coli* relies mainly on pathway overexpression on complex medium and some additional cofactor engineering to provide more NADPH [22]. However, advancing toward industrial-scale processes requires cheaper media without complex components such as yeast extract and a suitable strain and process design. The latter two hinge on a system-level mechanistic understanding of acetate-fueled cells producing isopropanol, which has yet to be established. Very little is known about the metabolic requirements for isopropanol formation, whether it can be realized growth-dependently or -independently and which measures are available to divert carbon flux toward Thl for efficient production. However, these factors need to be accommodated in process design, for instance, by applying different kinds of nutrient limitations or starvations in case a growth limitation is necessary.

Therefore, the aim of this study was to implement an efficient bioproduction scenario for isopropanol synthesis from acetate. To that end, we constructed a constitutively expressed pathway, tested it in several *E. coli* host strains, and designed a process considering inherent metabolic constraints. We evaluated different process modes and performed proteomics and acetylation analysis. Moreover, we carried out metabolic modeling with the physiological data to elucidate the driving forces and potential limitations behind efficient isopropanol production on acetate. Based on the fundamental understandings of isopropanol-producing *E. coli,* we propose strategies for further strain and process improvements which will enable successful large-scale process development.

## Materials and methods

### Bacterial strains and media

*E. coli* W (DSM 1116 = ATCC 9637 = NCIMB 8666) from DSMZ (Braunschweig, Germany), *E. coli* W *ΔldhA ΔadhE* (*E. coli* W KO2, kind gift of Prof. Michael Sauer (BOKU, Vienna, Austria)), and *E. coli* W *ΔldhA ΔadhE Δpta ΔfrdA* (*E. coli* W KO4, kind gift of Prof. Michael Sauer, BOKU, Austria) were used as a host organism to derive all strains used in this study. *E. coli* TOP10™ (Thermo Fisher Scientific (Thermo Fisher Scientific Inc. (NYSE: TMO)) was used for plasmid construction and propagation steps. A comprehensive list of all bacterial strains used and created in this study can be found in Table S1 in Additional file 1.

All chemicals were purchased from Carl Roth GmbH + Co. KG (Karlsruhe, Germany) if not stated otherwise. Liquid lysogeny broth (LB), prepared with 10 g L^−1^ peptone, 10 g L^−1^ NaCl and 5 g L^−1^ yeast extract, was used for all plasmid propagation steps during cloning, for precultures of shake flask experiments and the first preculture step for bioreactor cultivations. To prepare LB plates, 15 g L^−1^ of agar–agar was added. The base medium for shake flask main cultures, the second preculture step for bioreactor cultivations, and the batch medium for bioreactor cultivations were derived from Riesenberg et al. [27] at pH 7 containing: 13.3 g L^−1^ KH_2_PO_4_, 4.0 g L^−1^ or 1 g L^−1^ (NH_4_)_2_HPO_4_ (4 g L^−1^ yielded about 6.5 g L^−1^ biomass, 1 g L^-1^ yielded about 2 g L^−1^ biomass in the batches), 1.7 g L^−1^ citric acid (autoclaved) 1.2 g L^−1^ MgSO_4_ * 7 H_2_O, 0.10 g L^−1^ Fe(III)citrate, 0.0084 g L^−1^ EDTA, 0.013 g L^−1^ Zn(CH_3_COO)_2_ * 2 H_2_O, 0.0025 g L^−1^ CoCl_2_ * 6 H_2_O (Merck KGaA, Darmstadt, Germany), 0.015 g L^−1^ MnCl_2_ * 4 H_2_O, 0.0012 g L^−1^ CuCl_2_ * 2 H_2_O, 0.0030 g L^−1^ H_3_BO_3_, 0.0025 g L^−1^ Na_2_MoO_4_ * 2 H_2_O (sterile filtered). For shake flask main cultures, 5 g L^−1^ of sodium acetate was added as carbon source, the second bioreactor preculture step was carried out on 10 g L^−1^ glucose, and the bioreactors contained 10 g L^−1^ of sodium acetate for the batch. All LB and base media were supplemented with 50 mg L^−1^ of kanamycin (kan) or 100 mg L^−1^ carbenicillin (carb) as necessary.

The feed medium for the bioreactors was comprised of 150 g L^−1^ sodium acetate, 12.45 g L^−1^ MgSO_4_ * 7H_2_O, 0.025 g L^−1^ Fe(III)citrate, 0.0081 g L^−1^ EDTA, 0.01 g L^−1^ Zn(CH_3_COO)_2_ * 2 H_2_O, 0.0025 g L^−1^ CoCl_2_ * 6 H_2_O (Merck KGaA, Darmstadt, Germany), 0.015 g L^−1^ MnCl_2_ * 4 H_2_O, 0.0012 g L^−1^ CuCl_2_ * 2 H_2_O, 0.0030 g L^−1^ H_3_BO_3_, 0.0025 g L^−1^ Na_2_MoO_4_ * 2 H_2_O.

### Plasmid construction

Golden MOCS [28] based on Golden Gate cloning [29, 30] was used to assemble a plasmid containing the genes for the isopropanol production pathway. The genes for thiolase (*thl* from *Clostridium acetobutylicum*), acetoacetate decarboxylase (*adc* from *C. acetobutylicum*), NADPH-dependent secondary alcohol dehydrogenase (*adh* from *Clostridium beijerinckii*), and NADH-dependent secondary alcohol dehydrogenase (*adh1* from *Gordonia* sp. TY-5) were codon-optimized and ordered as gBlocks® from Integrated DNA Technologies (IA, USA), while *atoDA* was PCR amplified from *E. coli* MG1655 via Q5 High-Fidelity DNA Polymerase (New England Biolabs, MA, USA) using the primers listed in Table S3 (Additional file 1). All primers were purchased from Integrated DNA Technologies (IA, USA). Complete sequences of the codon-optimized genes are listed in Additional file 1.

Pathway assembly was performed as described previously using promoters BBa_J23114 (p114) or BBa_J23105 (p105) from the Anderson constitutive promoter library and BBa_B1001 as terminator [29, 30]. The correct integration into backbone 1 (BB1) was confirmed via Sanger sequencing. The correct assembly of the final constructs was verified via restriction digests. The plasmids with the completed pathway are listed in Table S2 (Additional file 1) and were transformed into *E. coli* W, *E. coli* W KO2 and *E. coli* W KO4 via electroporation. Frozen stocks of the strains were kept at − 80 °C in 20 % (w/v) glycerol. Depictions of the final four pathways on the plasmids are shown in Fig. S1 (Additional file 1).

### Preculture preparation

From the frozen stocks, cells were streaked onto LB-kanamycin plates and grown overnight at 37 °C. For the biological replicates, several single colonies were picked and used to inoculate 50 mL of LB in 500 mL shake flasks cultivated for approximately 8 h at 37 °C and 230 rpm.

For bioreactor cultivations, a second preculture step was carried out. Therefore, 200 mL of base medium in a 2 L ultra-high yield flask containing 10 g L^−1^ glucose was inoculated to 0.5 optical density at 600 nm (OD_600_) and the cells were kept at 37 °C and 230 rpm for 16 h. In this step, glucose was used as a carbon source to provide enough biomass for bioreactor cultivations in less than 24 h.

Afterward, the cells were transferred to sterile centrifuge beakers and centrifuged at 4000*g* for 5 min at 20 °C. The cells were washed with half the preculture volume of sterile 0.9% NaCl and centrifuged again. The supernatant was replaced by 5 mL (for shake flask experiments) or 10 mL (for bioreactor cultivations) of sterile 0.9 % (w/v) NaCl and the cells were resuspended. After verifying OD_600_, the shake flask main cultures or bioreactors were inoculated to an optical density of 0.5.

### Shake flask experiments

After inoculation, biological triplicates of each tested condition grew at 25 °C and 230 rpm for 72 h. Samples were taken every 24 h to determine optical density and quantify metabolites via HPLC measurements.

### Bioreactor cultivations

Bioreactor cultivations were carried out in four parallel DASGIP® Benchtop Bioreactors for Microbiology (Eppendorf AG, Hamburg, Germany) with 1 L working volume. The gassing rate with pressurized air was set to 30 L h^−1^ (0.5 vvm), while the temperature was kept at 25 °C or 37 °C. The pH was monitored by an EasyFerm Plus PHI K8 225 (Hamilton, Reno, NV, USA) electrode and maintained at 7 by adding 2 M KOH (to avoid introducing additional nitrogen) or 5 M H_3_PO_4_ via a MP8 multi-pump module (Eppendorf AG, Hamburg, Germany). Dissolved oxygen concentration was measured with a VisiFerm DO ECS 225 H2 probe (Hamilton, Reno, NV, USA) and kept above 30% by increasing stirrer speed (up to 1000 rpm) and increasing O_2_-concentration in the in-gas flow. Bioreactor off gas was led through three consecutive wash bottles filled with 400 mL dH_2_O on ice before being analyzed for O_2_ and CO_2_ by a GA4 gas analyzer module (Eppendorf AG, Hamburg, Germany). To avoid carbon depletion, an acetate feed was applied. The feed rate was adjusted manually to keep the acetate concentration between 2 and 8 g L^−1^.

Samples were taken regularly from the reactors and all wash flasks. Biomass formation was estimated by measuring OD_600_ in a spectrophotometer (Onda Spectrophotometer V-10 Plus, Giorgio Bormac s.r.l, Carpi (MO), Italy). After centrifugation of the cultivation broth (21,913 g, 10 min, 4 °C), concentrations of acetate and NH_4_^+^ in the supernatant were determined with a Cedex Bio HT Analyzer (Roche, Switzerland) to adjust the feed rate and determine the start of the nitrogen-starvation phase. To enable accurate further calculations, concentrations of acetate, lactate, pyruvate, formate, succinate, ethanol, acetone, and isopropanol were determined via HPLC measurement in the supernatant. To enable comparison, product titers are given as g L^−1^ or mmol L^−1^ bioreactor volume although a percentage of product was found in the wash flasks.

### Biomass determination

Dry cell weight (DCW) was measured gravimetrically at the end of each cultivation in triplicates. 5 mL of sample was centrifuged at 4000*g* for 10 min at 4 °C in pre-weighed glass tubes. Afterward, the cells were washed with 5 mL of filtered 0.9% NaCl. The supernatant was discarded, and the pellets were dried at 110 °C for 72 h. A correlation between OD_600_ and biomass concentration was established to calculate the biomass for each sample point.

### HPLC analysis

Alcohols and organic acids were determined on an Aminex HPX-87H column (300 × 7.8 mm, Bio-Rad, Hercules/CA, USA) in a Vanquish™ Core HPLC System (Thermo Scientific, Waltham/MA, USA). The mobile phase was 4 mM H_2_SO_4_, the flow rate set to 0.6 mL min^−1^ with a method run at 60 °C for 30 min.

Metabolites were detected via a refractive index detector (ERC RefractoMax 520, Thermo Scientific, Waltham/MA, USA) and an UV detector (Vanquish™ Variable Wavelength Detector Vanquish VWD-C, Thermo Scientific, Waltham/MA, USA) at 280 nm (acetone and pyruvate).

450 µL of sample was mixed with 50 µL of 40 mM H_2_SO_4_ and centrifuged at 21,913 g for 10 min at 4 °C. The supernatant was used for analysis. Standards were treated the same as the samples and a 6-point calibration curve was used for quantification between 0.05 and 10 g L^−1^ using an injection volume of 10 µL for all samples.

### Proteomics sampling

For shotgun proteomics and acetylated proteomics, biological quadruplicates of the respective bioreactor cultivations were performed. Samples containing approximately 0.0015 g (for shotgun proteomics) and 0.01 g (for acetylated proteomics) biomass were taken and immediately centrifuged at 21,913 g at 4 °C. The supernatant was carefully removed, and the pellets were snap frozen in liquid nitrogen. Frozen cells were stored at − 80 °C until further analysis.

### Proteomics sample preparation

#### Shotgun proteomics

Samples were lysed by sonication in 150–300 µL of lysis buffer (10 mM Tris (2-carboxyethyl) phosphine (TCEP), 40 mM chloroacetamide (CAA), 1% sodium dodecyl-sulfate, 100 mM Tris (pH = 8.5)) and 80 µg of protein was acetone precipitated overnight. After centrifugation, the resulting protein pellets were dissolved in 12.5% trifluoroethanol/Tris (TFE; pH = 8.5), diluted to 5% TFE and digested overnight with trypsin (1:50 = enzyme:protein ratio). Upon desalt, 300 ng of each sample was used for LC–MS/MS analysis.

#### Acetylated peptides

Bacterial pellets were lysed in 2 mL of the cold lysis buffer (20 mM HEPES (pH = 8), 7 M guanidine hydrochloride, 1 mM sodium orthovanadate, 2.5 mM sodium pyrophosphate, 1 mM β-glycerophosphate), and the lysates were cleared by centrifugation (20,000*g* for 15 min at 4 °C). Following protein estimation, 3.5 mg of proteins was reduced and alkylated using 10 mM TCEP and 40 mM CAA, respectively), then digested overnight with trypsin (1:20 = enzyme:protein ratio). Digests were purified on C18 Sep-Pak cartridges (Waters) according to the manufacturer’s instruction, prior to being subjected to immunoaffinity purification (IAP) of Ac-K peptides with PTM Scan Acetyl-Lysine Motif [Ac-K] Kit (Cell Signaling) following the supplemented protocol. Briefly, peptides were resuspended in 400 µL IAP buffer, cleared by centrifugation, then incubated with the antibody beads on an over-head rotator for 2 h at 4 °C. Beads were collected by centrifugation, washed, and the peptides were eluted using 100 µL of 0.15% TFA. Lastly, acetylated peptides were desalted using in-house-made C18 stage tips (Empore) and, upon desalt, resuspended in 10 µL of mobile phase A. 4 µL of the sample was used for injection and consequent LC–MS/MS analysis.

#### Proteomics LC–MS/MS analysis

Chromatography was carried out on an Ultimate 3000 RCS Nano Dionex system equipped with an Ionopticks Aurora Series UHPLC C18 column (250 mm × 75 µm, 1.6 µm) (Ionopticks). Solvent A was 0.1% formic acid in water and solvent B acetonitrile/0.1 % formic acid. Total LC–MS/MS run per sample was 86.5 min with the following gradient: 0–5.5 min: 2 % B; 5.5–25.5 min: 2–10 % B; 25.5–45.5 min: 10–25 % B, 45.5–55.5 min: 25–37 % B, 55.5–85.5 min: 37–80 % B; 65.5–75.5 min: 80 % B; 75.5–76.5 min: 80–2 % B; 76.5–86.5 min: 2 % B at a flow rate of 400 nL min^−1^ and 40 °C. The timsTOF mass spectrometer (Bruker Daltonics, Germany) was operated in positive mode with enabled trapped Ion Mobility Spectrometry (TIMS) at 100 % duty cycle (100 ms ramp and accumulation time). Source capillary voltage was set to 1400 V and dry gas flow to 3 L/min at 180 °C. Scan mode was set to parallel accumulation-serial fragmentation (PASEF) for the scan range of 100–1700 m/z. Precursor selection was based on their intensity (data-dependent acquisition) and the precursors were allowed to accumulate for a total of four ramps per PASEF cycle, bringing the total cycle time to 0.53 s.

#### Proteomics data processing

##### Shotgun proteomics

Raw data including protein annotation and database search were carried out in MaxQuant (version 2.0.3.0) [31, 32]. *E. coli* strain W fasta file obtained from the predicted protein NCBI database (txid: 566,546, 4660 entries, downloaded on 2022/06/13) was modified to include sequences for AtoA (atoD_ECO_MG1655), AtoD (atoD_ECO_MG1655), Adh_CBE and Adc_CAB_ATCC824_DSM79, and used for the database search. Methionine oxidation was set as variable and carbamidomethylation as fixed modification and up to two miss-cleavages were allowed for trypsin digestion. False discovery rate (FDR) for peptide spectral matching as well as for peptide and protein matching was set to 1%. Match between runs was enabled and the label-free protein quantitation was carried out through maxLFQ algorithm. The obtained list of quantified proteins was then imported into Perseus (v1.6.14.0) [33], log-transformed, and grouped per sample group ((25 or 37 °C) and time point of sample collection). Grouped samples were then pairwise filtered to have at least three valid values in at least one group and the missing values were imputed from the normal distribution prior to statistical analysis. This reduced the sample matrix from 2680 entries to 2062–2410 entries on which Student t-tests were carried out with the following cut-off: FDR corrected *p*-value < 0.05 and S0 0.1. In some cases, non-FDR corrected significant hits were considered.

When comparing within one cultivation temperature, the first sample point (early growth condition) taken at an OD_600_ of about 1 served as a reference condition. In comparisons between temperatures, equivalent sample times were compared with the data from 25 °C serving as a reference. For both approaches, all identified proteins of the TCA cycle or with a significant log2 fold change of ± 2 in at least one of the scenarios were considered for further analysis. These proteins were then grouped according to their metabolic function. The most significant groups for within- and between-temperature comparisons are shown in Figs. 4, 5, while the full versions of within- and between-temperature comparisons can be found in Figs. S5–S20 in Additional file 1.

##### Acetylation proteomics

Protein annotation and database search were carried out in Frag Pipe (version 19.0; MS Fragger version 3.7) [34] with an *E. coli* SwissProt fasta file containing 4357 inputs (downloaded on 25.01.2023). Specified protein modifications for the search were: methionine oxidation, N-terminal, and lysin acetylation as variable modifications and carbamidomethylation as fixed. The resulting modified peptide list (27,463 peptides) was then loaded in Perseus (v1.6.14.0) [33], log2 transformed and filtered to keep only acetylated peptides (9584 entries). The samples were again grouped and pairwise filtered to keep only those peptides with at least three reported valid values in at least one of the groups. This reduced the matrix to 1278–3010 acetylated peptides on which Student t-tests were carried out as aforementioned.

Due to the varying efficiency of sample preparation, it was not possible to compare individual sample points reliably. Instead, we compared the general acetylation of peptides in samples from 25 °C cultivations against the general acetylation in samples from 37 °C cultivations (volcano plot and principal component analysis plot available in Fig. S21 and Fig. S22 in Additional file 1). For further analysis, proteins with *p*-value significant peptides were grouped according to their general metabolic function and whether they exhibited more acetylation at 25 °C or 37 °C (details can be found in Additional file 2). Where the same peptide was detected multiple times, recalculation of the log2 fold change was done from the median intensities of all its detections. For both shotgun proteomics and acetylation proteomics, datasets including the raw data, search parameters as well identification/quantitation output were uploaded to the ProteomeXchange Consortium (http://proteomecentral.proteomexchange.org) via the PRIDE partner repository [35] with the dataset identifier PXD047939.

#### Metabolic flux estimation

To estimate flux distributions in the central carbon metabolism of *E. coli*, CNApy (Version: 1.1.8; [36]) was used with the core model ECC2comp [37] and the genome-scale model iJO1366 [38], respectively, where the reactions for isopropanol formation were added. Parsimonious Flux Balance Analysis [39], in short pFBA, was carried out with the objective function to maximize the non-growth-associated ATP maintenance energy (ATPM reaction flux). This is a reasonable objective function if the growth rate has been measured (and can, thus, not be optimized). Using pFBA ensures that this objective is maximized under the secondary objective to achieve this maximum with a minimal sum of absolute fluxes in the network to roughly reflect efficient enzyme usage by the cell. The specific rates for growth, acetate uptake, oxygen uptake, CO_2_, acetone, and isopropanol formation (obtained as means of the biological quadruplicates of the bioreactor cultivations) were used as given (fixed) flux values for the pFBA. To enable modeling of intracellular fluxes in an acetate-based cultivation scenario, other carbon source uptakes (e.g., glucose) were deactivated during model simulations. Additionally, known futile cycles were disabled by setting certain fluxes to zero, which included acetate export, ubiquinone pyruvate dehydrogenase (PoxB), succinate transmembrane transport, and fumarate reductase (Frd). In a similar manner, fluxes of export reactions of side products which were not detected in the supernatant were assumed to be zero (H_2_, lactate, formate, and ethanol).

The feasibility of the flux scenarios was assessed with a demanded minimal ATPM reaction flux of 3.15 mmol gDW^−1^ h^−1^. If a given set of fixed fluxes led to an infeasible pFBA scenario, CNApy’s feasibility algorithm was used to perform minor adjustments to the input rates using a linear program for minimizing the sum of deviations from the given fluxes [40]. pFBA was then performed with the set of feasibility-corrected flux values. In most cases, the feasibility-corrected values were within the standard deviation of the original input values. A complete list of the resulting flux scenarios is denoted in an additional file (Additional file 3). A visual representation of a relevant flux scenario in the ECC2comp model computed with CNApy can be found in Fig. S23 in Additional file 1.

## Results and discussion

### Initial strain design and limitations

To enable isopropanol production on acetate, we opted for a plasmid-based expression of the isopropanol pathway. We made use of constitutive promoters of varying strength to be independent from expensive inducers and to better control pathway expression. To that end, individual expression cassettes for each step of the pathway were combined. Additionally, two versions of the pathway were constructed that differed in the cofactor (NADPH or NADH) for the final reduction step from acetone to isopropanol. To that end, the NADH-dependent *adh1* from *Gordonia* sp. TY-5 instead of the *adh* from *Clostridium beijerinckii* was used. We reasoned that the higher availability of NADH compared to NADPH (2 instead of 1 mol are generated in the TCA cycle per acetate utilized) might enable a more efficient conversion of acetone into isopropanol.

As host strains, we chose *E. coli* W for its acetate tolerance as well as *E. coli* W KO2 (Δ*adhE* Δ*ldhA*) and *E. coli* W KO4 (Δ*adhE* Δ*ldhA* Δ*pta* Δ*frdA*) because we could previously show that deletions in mixed acid fermentation pathways are beneficial for aerobic acetate-based 2,3-butanediol production [5].

### Strain characterization

**Fig. 2:**
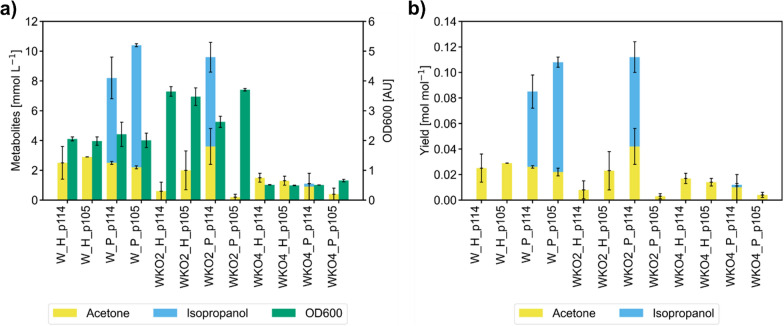
Summary of the strain screening results of the isopropanol strains. Bars and error bars denote means obtained from biological triplicates and their standard deviation. Strains: W = *E. coli* W, WKO2 = *E. coli* W KO2 (see “Bacterial strains and media” section), WKO4 = *E. coli* W KO4 (see “Bacterial strains and media” section). H = construct containing NADH-dependent *adh1* from *Gordonia* sp. TY-5, P = construct containing NADPH-dependent *adh* from *C. beijerinckii*. Promoters: BBa_J23114 from Anderson promoter library = p114; BBa_J105 from Anderson promoter library = p105. **a** Product titers and optical density at the end of the screening cultivations. **b** Yields of acetone and isopropanol per acetate for each strain

To assess isopropanol production and determine the effects of the different constructs we conducted screening experiments in shake flasks. A temperature of 25 °C was chosen to avoid excessive product loss via evaporation. Titers and yields are summarized in Fig. 2. More detailed data of the shake flask experiments can be found in Figs. S2–4 (Additional file 1).

All tested strains were able to produce acetone to some extent, with the wild-type strain background reaching about 2–3 mmol L^−1^ of acetone, the KO4 strains ranging between 1 and 2 mmol L^−1^ of acetone and the KO2 strains varying from 0.2 to 4 mmol L^−1^. Both wild-type strains carrying the NADPH-dependent pathway as well as *E. coli* WKO2 P_p114 produced significant amounts of isopropanol, whereas no isopropanol was formed when the pathways were tested in the KO4 knock-out strain. Although deletions in mixed acid fermentation pathways were beneficial for 2,3-butanediol production from acetate [5], the knock-out strain backgrounds had a negative effect on isopropanol formation. The reason for this difference might be the different branching points of the pathways for 2,3-butanediol (pyruvate) and isopropanol (acetyl-CoA).

Isopropanol formation was also absent in strains containing the NADH-dependent version of the pathway. Consequently, we selected *E. coli* W P_p105 as the best isopropanol-producing strain for further characterization as it showed the highest isopropanol titer and yield per acetate as well as a high isopropanol:acetone ratio (Fig. 2).

We note that the cells showed growth-decoupled product formation as isopropanol synthesis was most efficient during an initial 24-h lag phase on acetate and significantly decreased during growth. This fact was subsequently taken into account when designing a suitable bioprocessing strategy.

### Process design

To realize a growth-decoupled production process, which appeared to be a prerequisite for efficient isopropanol production, we tested limiting the cells’ carbon and nitrogen supply. A carbon limitation proved unsuitable, because the cells heavily favored growth over product formation under carbon limiting conditions (Fig. S24 in Additional file 1). Instead, keeping the acetate concentration between 30 and 170 mmol L^−1^ seemed best to enable isopropanol production. Nitrogen limitation in contrast proved to be a more suitable alternative. Although preliminary experiments using a nitrogen limiting feed did not support isopropanol formation but led to increased biomass formation (Fig. S25 in Additional file 1), a complete N-starvation phase after growth was able to successfully trigger product formation.

The cultivation temperature was identified as another major influence factor for the process design. We reasoned that lower temperatures might mitigate stress caused by acetate and product formation, whereas higher temperatures could enable faster processes by accelerating growth and metabolism. Consequently, the final process encompassed an N-excess phase for growth and an N-starvation phase for production with a surplus of acetate either operated at 25 °C or 37 °C.

### Physiological characterization at different temperatures in bioreactors

**Fig. 3:**
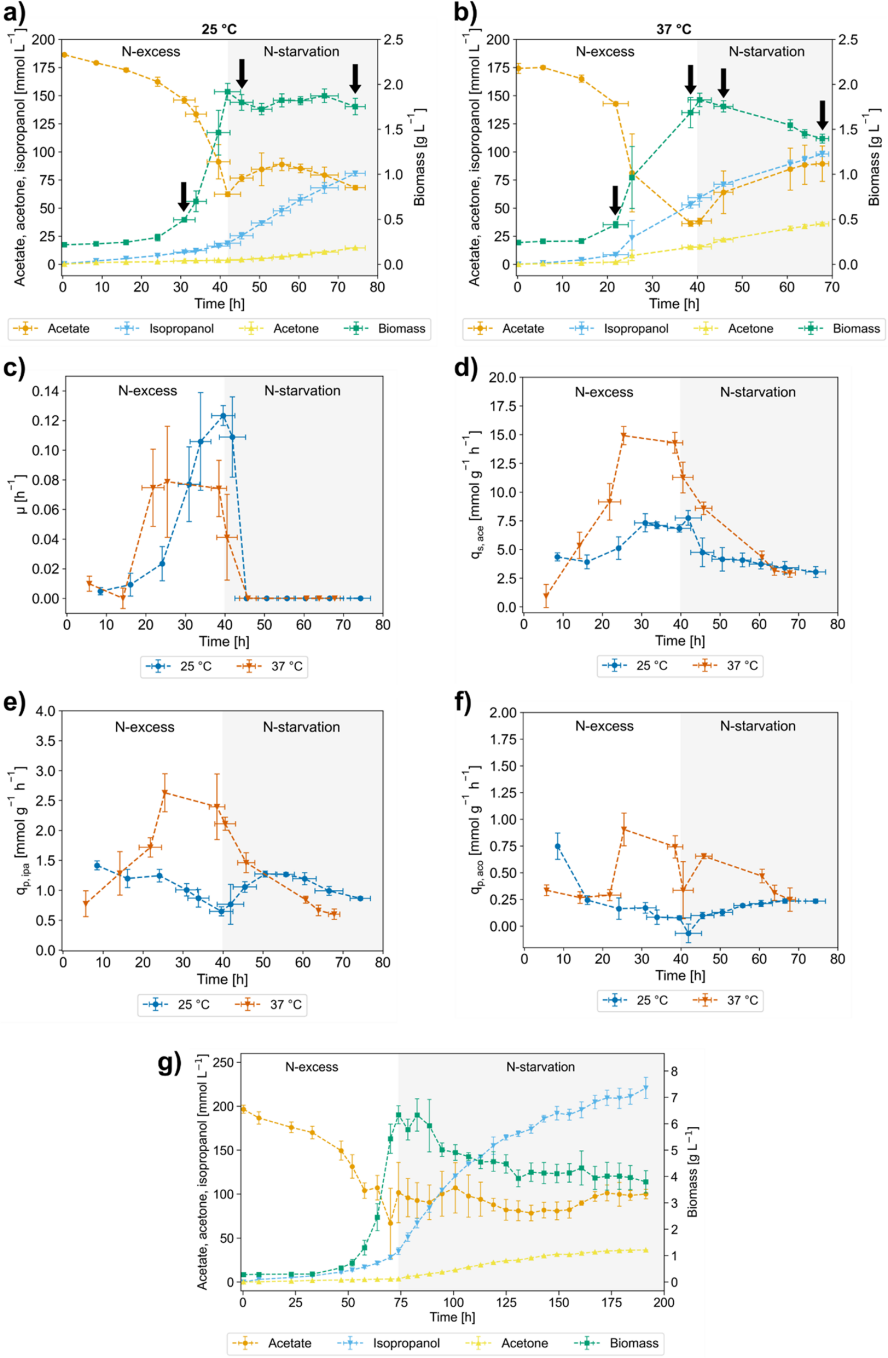
Physiological data gained from cultivation of *E. coli* IPA_P_p105 at 25 °C and 37 °C. Error bars show the standard deviation of 4 independent biological quadruplicates (or duplicates in figure g). **a** Concentration profiles of acetate, isopropanol, acetone and biomass at 25 °C. Black arrows denote -omics sampling. **b** Concentration profiles of acetate, isopropanol, acetone and biomass at 37 °C. Black arrows denote -omics sampling. **c** Specific growth rates (µ) at 25 °C and 37 °C. **d** Specific acetate uptake (q_s,ace_) at 25 °C and 37 °C. **e** Specific isopropanol production rates (q_p,ipa_) at 25 °C and 37 °C. **f** Specific acetone production rates (q_p,aco_) at 25 °C and 37 °C. **g** Concentration profiles of acetate, isopropanol, acetone and biomass in a high-density 2-stage bioprocess with extended N-starvation phase

For the growth phase of the process, we used 1 g L^−1^ (NH_4_)_2_HPO_4_ as nitrogen source which we expected to yield a final biomass concentration of about 2 g L^−1^. The initial acetate concentration was set to ~ 175 mmol L^−1^ and maintained above 30 mmol L^−1^ by continuous feeding (Fig. 3a, b).

After a lag phase of 15–25 h, the cells grew up to 2 g L^−1^ of biomass as long as nitrogen was available (Fig. 3a, b). Following the depletion of NH_4_^+^ isopropanol and acetone were formed. After 30–35 h of N-starvation, titers of 80.7 ± 2.2 mmol L^−1^ (4.85 ± 0.13 g L^−1^) isopropanol after 75 h at 25 °C and 98.1 ± 2.4 mmol L^−1^ (5.90 ± 0.14 g L^−1^) isopropanol after 70 h at 37 °C were observed. It is important to note that only about 60–70% of the overall isopropanol was found in the reactor while the remaining 30–40 % were stripped from the reactor.

Despite a higher titer and shorter lag phase at 37 °C, we found that our process strategy for growth-decoupled production using N-starvation worked better at 25 °C. At low temperature and excess NH_4_^+^ available, the cells favored growth and only produced marginal amounts of isopropanol and acetone. N-starvation triggered a total growth stop and an increase of the specific isopropanol formation rate, and most of the isopropanol was produced in the N-starvation phase (Fig. 3a).

At 37 °C, growth slowed around 30 h already, and production started early while excess nitrogen was still present (Fig. 3b, c and e) and approximately 50% of product originated from the N-excess phase. The stunted growth was accompanied by a significant rise in the specific acetate uptake rate, which made it necessary to add an acetate feed already during the N-excess phase to avoid acetate depletion (Fig. 3d). Nevertheless, subsequent nitrogen depletion also led to a complete growth stop and isopropanol production continued during the N-starvation phase. Regardless of temperature, N-starvation caused a significant drop in the specific acetate uptake which also negatively affected product formation rates. In further experiments, we attempted to mitigate this effect by resupplying small amounts of nitrogen for the cells to recover their metabolic activity (Fig. S26 in Additional file 1). However, adding nitrogen after about 45 h of starvation did not reinitiate growth or boost acetate uptake.

Toward the end of the processes, the product ratio shifted from isopropanol toward acetone, potentially due to insufficient NADPH supply for acetone conversion.

Comparing the overall process performance between 25 °C and 37 °C, carbon is more efficiently used at lower temperatures. At 25 °C, the cells exhibited a higher biomass and isopropanol yield and produced less CO_2_ compared to 37 °C (Table 1). With more biomass (~ 6.5 g L^−1^ biomass obtained from 4 g L^−1^ (NH_4_)_2_HPO_4_), the titer of the 25 °C-process could even be raised to 134 mmol L^−1^ (8.07 g L^−1^) isopropanol after 30 h of N-starvation, which corresponds to an increase of 66 % compared to the low-biomass process. After 115 h of N-starvation phase with ~ 6.5 g L^−1^ biomass 221 mmol L^−1^ (13.3 g L^−1^ = 274 % of the low-biomass process) isopropanol were achieved (Fig. 3 g).

**Table 1:** Yields (biomass (BM), isopropanol (IPA), acetone (ACO), and CO_2_ per acetate (ACE)) from low-density biological quadruplicates

Phase	Y_BM/ACE_ [g g^−1^]	Y_IPA/ACE_ [mol mol^−1^]	Y_ACO/ACE_ [mol mol^−1^]	Y_CO2/ACE_ [mol mol^−1^]
Growth 25 °C	0.221 ± 0.011	0.148 ± 0.010	0.027 ± 0.005	0.828 ± 0.020
Growth 37 °C	0.087 ± 0.005	0.189 ± 0.006	0.051 ± 0.005	0.943 ± 0.049
Production 25 °C	n. a.	0.262 ± 0.017	0.045 ± 0.011	0.958 ± 0.058
Production 37 °C	n. a.	0.187 ± 0.009	0.092 ± 0.006	1.088 ± 0.056
Overall 25 °C	0.088 ± 0.014	0.235 ± 0.030	0.041 ± 0.006	0.908 ± 0.017
Overall 37 °C	0.043 ± 0.001	0.188 ± 0.005	0.068 ± 0.003	1.003 ± 0.050

The isopropanol yield after 30 h of N-starvation in the low-biomass process at 25 °C amounted to 0.262 mol mol^−1^, which corresponds to about 75 % of the energy-balanced theoretical yield (0.35 mol mol^−1^) [9].

While a higher temperature accelerated the process and increased the isopropanol titer, it also rendered the process less predictable and less carbon efficient (Table 1). Consequently, only 54% of the energy-balanced theoretical yield were achieved at 37 °C.

### Proteome analysis

**Fig. 4:**
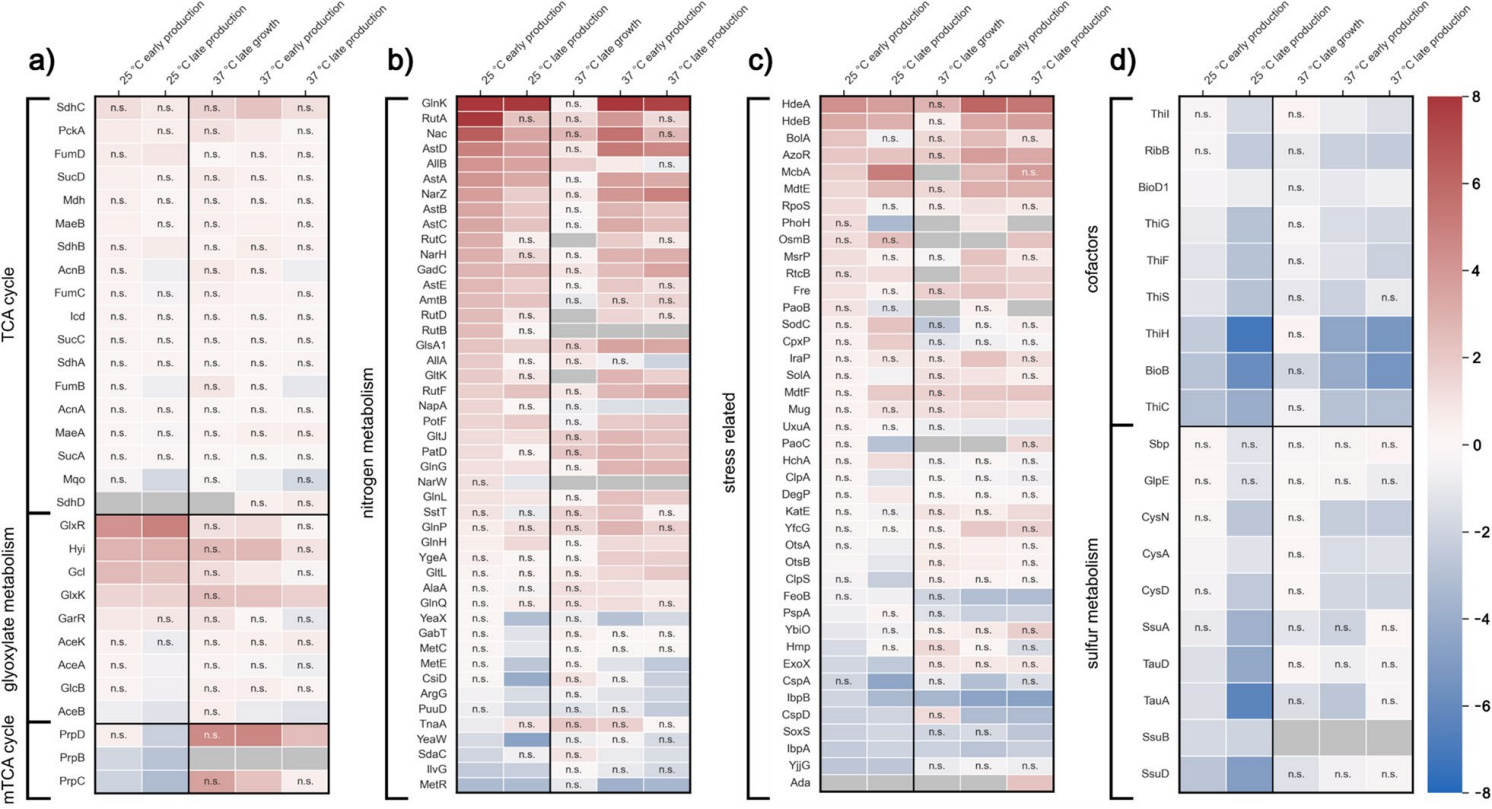
Within-temperature log2 fold changes of proteins. Proteins are grouped according to their function in the metabolism and results are given relative to the first -omics sample of the respective temperature. “n.s.” (“not significant”) indicates a non-significant fold change. Gray tiles denote that the protein was not detected in the sample. **a** Fold changes of the detected proteins assigned to the TCA cycle, the glyoxylate metabolism and the methyl-TCA cycle. **b** Fold changes of proteins assigned to nitrogen metabolism. **c** Fold changes of proteins assigned to stress-related processes. **d** Fold changes of proteins assigned to cofactor formation and sulfur metabolism

**Fig. 5:**
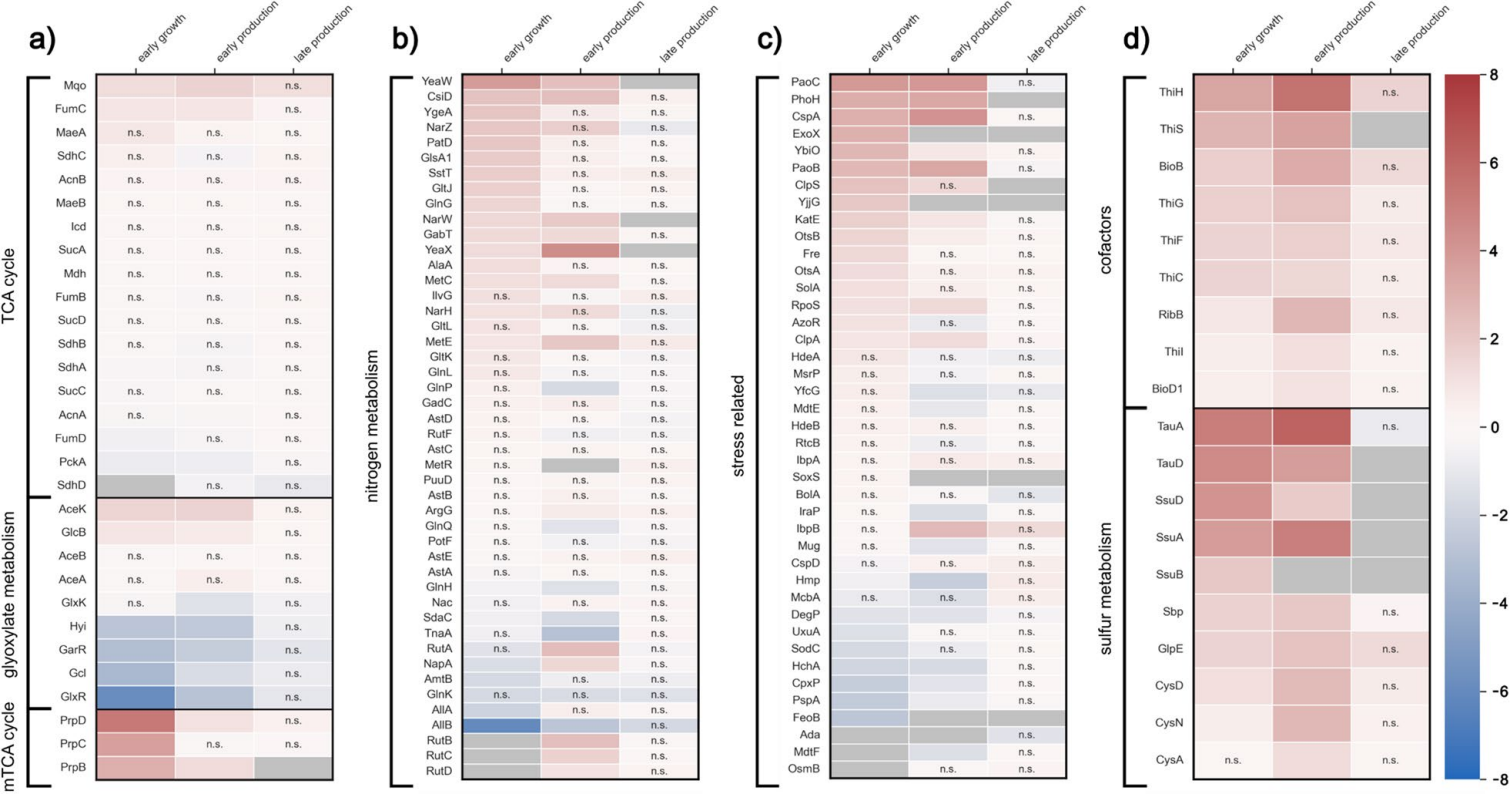
Across-temperature log2 fold changes of proteins. Proteins are grouped according to their function in the metabolism. For every compared phase between the different temperature processes the 25 °C sample was used as a reference (therefore red and blue color indicates a higher abundance at 25 °C and 37 °C, respectively). “n.s.” (“not significant”) indicates a non-significant fold change. Gray tiles denote that the protein was not detected in the compared sample. **a** Fold changes of the detected proteins assigned to the TCA cycle, the glyoxylate metabolism and the methyl-TCA cycle. **b** Fold changes of proteins assigned to nitrogen metabolism. **c** Fold changes of proteins assigned to stress-related processes. **d** Fold changes of proteins assigned to cofactor formation and sulfur metabolism

Next, we analyzed the proteome to better understand cell physiology and metabolic behavior of the different production scenarios, both during the process as well as in-between processes. To that end, we investigated behavior during growth (N-excess) as well as during the early and late production phases (N-starvation) at 25 and 37 °C (Figs. 4, 5). Using the data from the shotgun proteomics analysis, we compared relative changes between samples.

The TCA cycle and the anaplerotic glyoxylate shunt are central metabolic pathways with high importance for providing ATP, NADPH and precursors for growth when acetate serves as the sole carbon and energy source. However, on a proteome level, the TCA cycle seemed to be very stable as no significant differences in protein abundance were observed. One notable exception is the increase of enzymes catalyzing reactions around tartronate semialdehyde, a pathway branching from glyoxylate (Fig. 4a; glyoxylate carboligase–Gcl, hydroxypyruvate isomerase–Hyi, 2-hydroxy-3-oxopropionate reductase–GlxR and glycerate-3-kinase–GlxK). However, subsequent metabolic modeling of the data with the genome-scale model iJO1366 [38] revealed that even higher relative changes of fluxes in these reactions do not have a significant impact on central metabolic fluxes (data not shown).

Interestingly, the enzymes of the product pathway also did not show any significant change in abundance over time and the different conditions (Fig. S8 and Fig. S16 in Additional file 1).

Next, we analyzed the proteome related to nitrogen metabolism, where we expected to see a clear response to starvation. Our data confirmed that regulatory proteins like GlnK (nitrogen regulatory protein GlnK), GlnG (DNA-binding transcriptional regulator NtrC) and Nac (nitrogen assimilation regulatory protein nac) were upregulated relative to early growth conditions. Enzymes responsible for procuring nitrogen via degradation (e.g., AST-pathway enzymes for arginine) were also substantially more abundant.

On the other hand, the presence of enzymes related to amino acid formation (especially methionine) declined significantly during N-starvation (e.g., transcriptional regulator MetR, cystathionine beta-lyase MetC). As a result, replacing damaged proteins might become increasingly difficult for cells, which is also reflected in the reduced overall metabolic activity during prolonged N-starvation.

In addition to a direct response to N-starvation, we also observed a response in proteins related to stress. The proteins with the highest relative fold changes in the N-starvation phase were the acid stress chaperones HdeA and HdeB (Fig. 4c). It is, therefore, likely that N-starvation exacerbates acetate toxicity to which the cells react by producing larger amounts of chaperones to maintain homeostasis. Apart from general stress regulators like the RNA polymerase sigma factor (RpoS) and a DNA-binding transcriptional regulator (BolA), other upregulated proteins in the N-starvation phase were associated with osmotic stress (OsmB—osmotically inducible lipoprotein B), thiol-specific stress (AzoR—FMN-dependent NADH:quinone oxidoreductase), DNA/RNA-repair (RtcB—RNA-splicing ligase RtcB) and aldehyde stress (PaoB—Aldehyde oxidoreductase FAD-binding subunit). Presumably, the absence of nitrogen in addition to the presence of acetate causes a complex stress profile for the cells that requires different responses.

Beyond nitrogen metabolism and stress response, the N-starvation had considerable influence on enzymes involved in cofactor synthesis. While less pronounced at 25 °C, enzymes for biotin and thiamine biosynthesis experienced a notable downregulation (Fig. 4d). The lack of such essential cofactors may also play into the cells’ reduced metabolic activity toward the end of the process.

Additionally, all three systems for sulfur uptake were downregulated: the Ssu-transporter system, the sulfate assimilating enzymes CysA, CysD and CysN and the alkanesulfonate transporter proteins TauA and TauD. *E. coli* usually takes up sulfate via the CysAWTP complex, while the *tauABCD* and *ssuEADCB* genes are only expressed when the cells are starved for sulfate [41, 42]. However, a significant surplus of sulfur was present in the cultivation medium. We, therefore, hypothesize that sulfur uptake may be impaired due to the relatively high ATP demand for the transport or a decreased demand for sulfur due to the N-starvation prohibiting growth and decreasing general metabolic activity. Since sulfur is also needed for thiamine biosynthesis, an inhibited sulfur assimilation may be linked to the reduced thiamine formation.

### Proteome acetylation analysis

Acetylation is usually linked with changing substrate- or cofactor-affinity of enzymes and with regulatory mechanisms potentially exerting major influence on the central carbon metabolism. It can occur non-enzymatically, in which case acetyl-phosphate acts as the main acetyl-donor [43–45]. Since acetyl-phosphate is a key intermediate in acetate uptake, it plays a significant role in acetate-grown cells and acetate-based isopropanol production [46].

Comparing our processes at different temperatures, significantly more acetylated proteins seemed to be more prevalent at 37 °C (165 peptides, 56 proteins) than at 25 °C (21 peptides, 12 proteins) with a considerable number of them found either in the TCA cycle, associated with glyoxylate metabolism, glycolysis or lipid metabolism. However, the three most relevant targets in the context of isopropanol production are: (1) GltA, the entry point to the TCA cycle and a direct competitor of the product pathway for carbon flux, (2) ICDH, the main source of NADPH required for acetone to isopropanol reduction, and (3) isocitrate lyase (AceA), the entry point to the glyoxylate shunt which regulates cell growth.

We did not detect any differential acetylation for AceA. For GltA and ICDH, there are two known acetylation sites each which change enzyme activity: K283 (increase) and K295 (decrease) for GltA and K55 (increase) and K350 (increase) for ICDH [46, 47]. Of these sites, we found K55 in ICDH significantly more acetylated at 37 °C, which implies a higher activity potentially resulting in a higher NADPH availability per acetate. Other identified acetylation sites in GltA and ICDH seem to be located more toward the protein surface, away from active sites.

Nonetheless, increased acetylation at 37 °C in general might be linked to elevated stress levels. Since we also found a significant number of more acetylated proteins associated with stress response or refolding, this might point to less efficient stress mitigation at higher temperatures. However, additional work is required to determine the exact effects and underlying regulatory mechanisms.

### The potential “motor” of isopropanol formation

To further elucidate the cells’ behavior, we used parsimonious Flux Balance Analysis (pFBA; see Methods) and explored the intracellular carbon flow in the central metabolism for both temperatures tested and all sample points (Fig. 3a, b). We set the non-growth-associated maintenance energy (ATPM reaction flux) as the primary objective function for optimization in the pFBA simulations (see Methods ). ATPM represents a pseudo reaction quantifying the amount of metabolized ATP that is either used in non-growth-associated processes or for growth-related ATP requirements that were not captured in the biomass pseudo reaction in the stoichiometric model (see below). By summing up the amount of ATP generated by all ATP-producing reactions, we also determined the ATP demand of the cell for each scenario, which includes the ATP needed for ATPM and growth.

The scenarios were generally split into high cellular resource demand conditions in the growth phase and low cellular resource demand conditions in the N-starvation phase. The model predicted that during the high-demand conditions between 40 and 60 % of the total carbon from acetyl-CoA entered the TCA cycle via citrate synthase to produce NADPH (via isocirate dehydrogenase) and ATP (directly via succinyl-CoA ligase and indirectly via NADH used for respiratory ATP synthesis), both needed for growth. Between 5 and 20 % of carbon entered the TCA cycle via the anaplerotic glyoxylate shunt to provide carbon precursors for growth. In contrast, during low-demand conditions, much less acetate is taken up of which only 30–40 % entered the TCA cycle (almost exclusively via citrate synthase) while 60–70 % were used for IPA and ACO synthesis.

In a next step, we defined the Thl:TCA flux ratio, the isopropanol:acetone synthesis ratio (IPA:ACO) and the Thl:ICDH flux ratio as the key nodes which influence isopropanol production. The Thl:TCA ratio determines the carbon split between the product pathway and the central carbon metabolism, the IPA:ACO ratio describes the effectiveness of acetone conversion to IPA, and the Thl:ICDH ratio shows the availability of NADPH in relation to the product pathway flux.

**Fig. 6:**
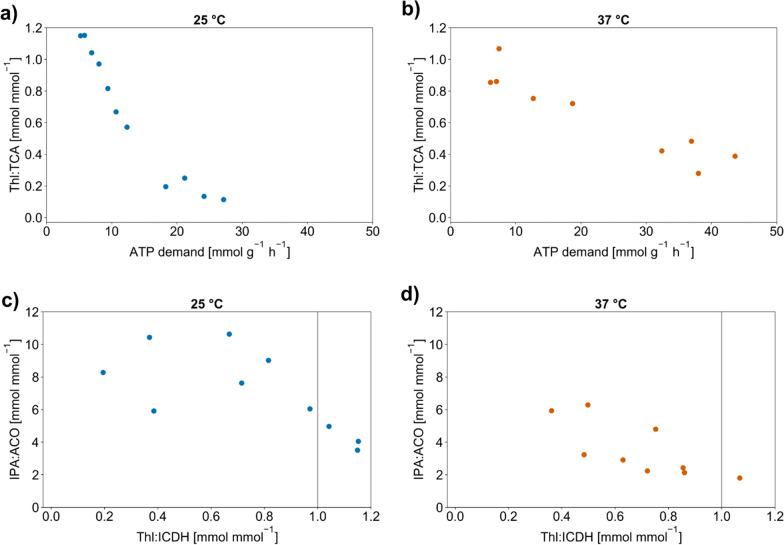
ATP demand and flux ratios at key nodes in the central carbon metabolism. **a** Thl:TCA flux ratio vs. ATP demand at 25 °C. **b** Thl:TCA flux ratio vs. ATP demand at 37 °C. **c** IPA:ACO flux ratio vs Thl:ICDH flux ratio at 25 °C. **d** IPA:ACO flux ratio vs Thl:ICDH flux ratio at 37 °C. A Thl:ICDH flux ratio of 1 represents the theoretical value for a perfect balance between NADPH provided by ICDH and NADPH needed for complete acetone conversion, which is indicated by the gray lines in c and d

First, we wanted to find out whether the total ATP demand influenced the amount of product formed. Therefore, we plotted the Thl:TCA flux distribution against the total amount of ATP produced (ATP demand) and found a negative correlation for both temperatures (Fig. 6a, b). At a high ATP demand, acetyl-CoA is mainly (40–60 %) directed toward the TCA cycle via the citrate synthase to fuel ATP (and NAD(P)H) production, while a smaller fraction of about 5–20 % enters the TCA cycle via the glyoxylate shunt needed to fuel carbon precursor pools. This limits the isopropanol pathway fluxes. When the demand of ATP and carbon is low, the flux through the TCA cycle decreases. As a result, carbon is directed toward the isopropanol production pathway (starting at Thl). Our investigation of NADPH supply revealed that for most conditions the amount of NADPH produced by ICDH exceeds the cellular requirements. As a result, surplus NADPH is partially used for IPA synthesis and by the transhydrogenase converting NADPH to NADH. Hence, we consider NADPH, at least stoichiometrically, not limiting for IPA synthesis, while the cellular demand for ATP and carbon precursors negatively affects isopropanol formation.

Interestingly, the shift toward carbon entering the isopropanol pathway at 25 °C occurs within a narrow range of ATPM fluxes (between 5 and 10 mmol g^−1^ h^−1^) whereas the shift range at 37 °C is wider (ATPM fluxes between 5 and 25 mmol g^−1^ h^−1^) (Fig. S27, Additional file 1). We identified two possible explanations for this behavior. First, ATPM fluxes could be increased at higher temperatures due to elevated stress levels, which we discovered via proteome analysis (e.g., increased chaperone activity; c.f. “Proteome analysis” and “Proteome acetylation analysis” sections). Second, the actual growth-dependent ATP demand could be higher than its representation in the metabolic model. We found a correlation between ATPM fluxes and the specific growth rates for both temperatures (Fig. S27, Additional file 1). At 25 °C, there is only a weak correlation which could be explained as growth-associated processes leading to more stress and non-growth-associated processes (e.g., protein refolding). However, the stronger correlation at 37 °C more likely indicates a certain underrepresentation of growth-associated ATP fluxes.

### The delicate balance between NADPH and product formation

The availability of NADPH as the electron donor for the reduction of acetone is another crucial factor for isopropanol formation. Using acetate as the substrate, the main source of NADPH generation is the isocitrate dehydrogenase (ICDH) reaction with little or no contribution from other reactions such as the pentose phosphate pathway generating NADPH during growth on glucose. Insufficient supply of NADPH can, therefore, lead to a shift from isopropanol to acetone production which we observed during the fermentation upon prolonged exposure of cells to N-starvation. Consequently, we explored NADPH availability as a key parameter for isopropanol selectivity using the IPA:ACO ratio in combination with the Thl:ICDH flux ratio (Fig. 6c, d). Generally, a Thl:ICDH flux ratio of 1 would theoretically allow for NADPH-balanced isopropanol formation; whereas, a ratio above 1 is expected to shift product formation toward acetone.

For both temperatures, a negative correlation between Thl:ICDH and IPA:ACO can indeed be observed. Consequently, more acetone is produced instead of isopropanol as Thl:ICDH approaches 1 (Fig. 6c, d). On the other hand, how much flux can enter the TCA cycle to fuel ICDH is again also dependent on ATP (and acetyl-CoA) demand for growth. At high-demand conditions during growth, more carbon is directed toward the TCA cycle, providing excess NADPH that can be used for isopropanol formation but at the same time limiting available acetyl-CoA for the product pathway. At low-demand conditions, less carbon enters the TCA cycle. Instead, it is directed toward the isopropanol pathway at the cost of less NADPH being produced via ICDH, which shifts the product balance toward acetone.

Overall, the effect was more pronounced at 37 °C where even at a lower Thl:ICDH ratio production leaned more toward acetone, even though NADPH was not considered stoichiometrically limiting (see “The potential “motor” of isopropanol formation” section). Reactions competing for NADPH such as the transhydrogenase reaction catalyzed by UdhA (soluble pyridine nucleotide transhydrogenase) could explain this behavior. pFBA reveals that at 25 °C about 25 % of NADPH from the ICDH is used by the transhydrogenase, while 45 % of NADPH are diverted at 37 °C. Therefore, even at a Thl:ICDH-flux ratio of about 0.5 mmol mmol^−1^, the product balance has already shifted toward acetone.

A comparison of the kinetic data of UdhA from *E. coli* (K_M_ of 68.29 µM for NADPH) and the secondary alcohol dehydrogenase from *C. beijerinckii* (K_M_ of 22 µM for NADPH) shows that the competition observed in the model data could be attributed to their similar affinities for NADPH [48, 49]. Even though there is no conclusive enzymatic data available at 25 °C, the model suggests that competition is less pronounced at lower temperatures.

### The two metabolic states of growth-decoupled isopropanol formation

**Fig. 7:**
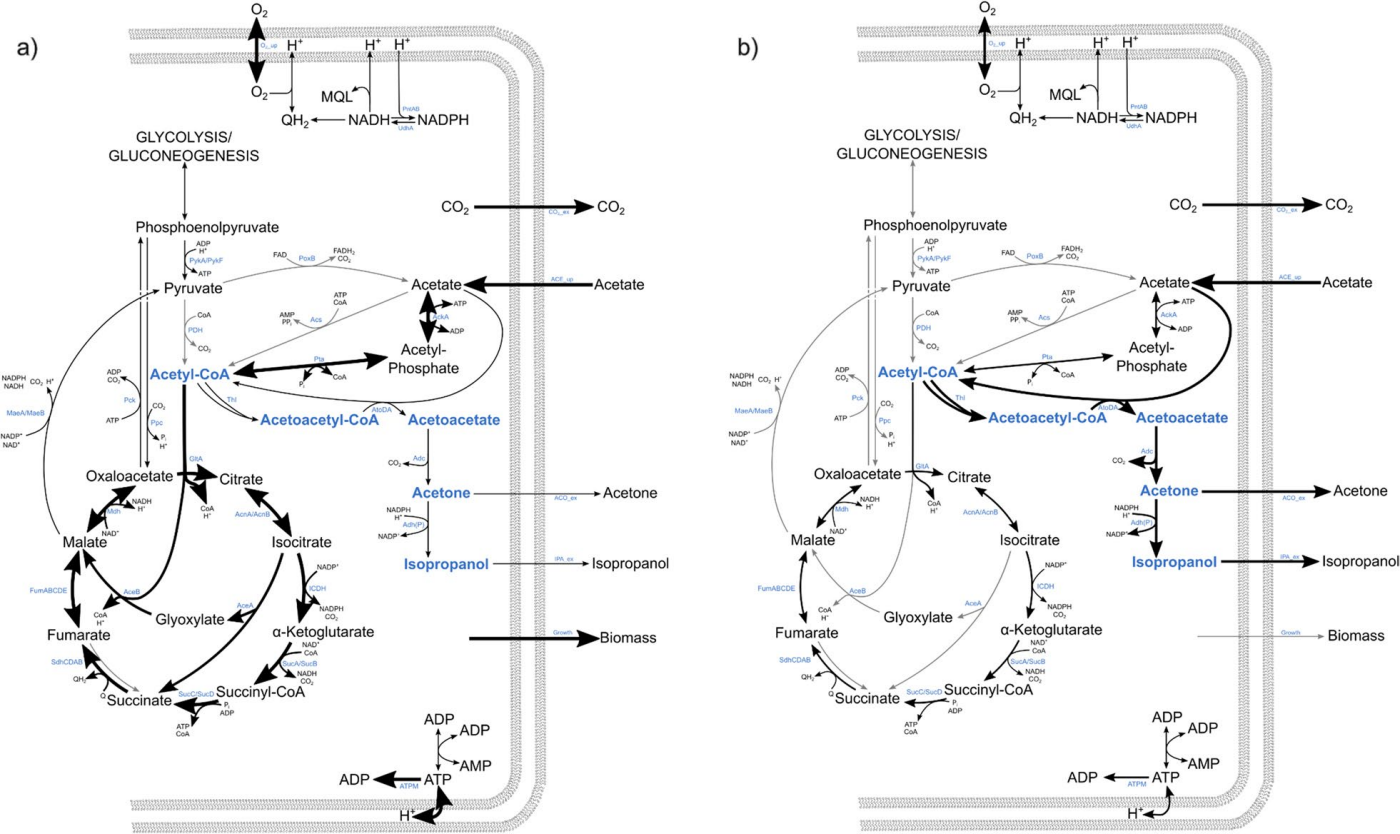
Metabolic scenarios of isopropanol-producing cells on acetate. Arrow thickness indicates flux level. Gray arrows denote fluxes that are considered (close to) zero. **a** Growth/high-resource-demand scenario during N-excess: Acetate is predominantly taken up via AckA and Pta and metabolized in the TCA cycle. Biomass formation is preferred while product formation remains low. **b** Production/low-resource-demand scenario during N-starvation: Acetate is taken up via AckA, Pta and AtoDA. A smaller amount of it enters the TCA cycle where the glyoxylate shunt is inactive due to a lack of biomass formation. Instead, acetyl-CoA is directed toward isopropanol formation. The ratio between Thl- and ICDH-flux determines the IPA:ACO ratio

From the modeling and physiological data, we were able to derive two general metabolic states of *E. coli* in the growth-decoupled isopropanol production process on acetate (Fig. 7).

During the N-excess phase, the metabolic flux distribution closely resembles Fig. 7a, where most acetate is directed toward the TCA cycle and growth with high ATP and carbon precursor demand. This state appears to be relatively stable as long as nitrogen is present, since high ATP demand feeds back to more flux through the TCA cycle. Product formation is generally low despite sufficient NADPH produced by ICDH, because carbon is directed toward the TCA cycle instead.

During N-starvation conditions, the growth stop leads to a lower ATP and carbon precursor demand (Fig. 7b) and the flux in the TCA cycle decreases. Since neither carbon nor energy are used for growth, we hypothesize that ATP or/and acetyl-CoA reach high concentrations limiting further acetate uptake. Isopropanol production constitutes a way for the cells to divert some acetyl-CoA away from the TCA cycle, which also reduces the ATP surplus, therefore it is favored under these conditions. Isopropanol can be produced efficiently if the remaining TCA cycle flux provides enough NADPH for the alcohol dehydrogenase, otherwise acetone accumulates instead. Due to the lack of biomass formation, there is no flux through the glyoxylate shunt which further reduces the oxygen demand. Eventually, the prolonged N-starvation leads to a general decrease of metabolic activity because damaged enzymes cannot be replaced and essential vitamin production declines, as underlined by decreasing rates for substrate uptake, product formation and CO_2_ production.

### Further metabolic engineering strategies for isopropanol and acetone production

Our investigations of strain requirements, process design and metabolic states fueling isopropanol production show that the main issues lie in the mode of production. Since acetyl-CoA is the branching point of the current heterologous isopropanol pathway, production and growth are bound to compete. Therefore, significant isopropanol formation only occurs in a growth-decoupled process design, where high concentrations of ATP and acetyl-CoA can be expected.

However, we have identified potential engineering approaches which could benefit such a process design. The first approach is to control ATP availability and demand, which we identified as the main driving force to direct carbon toward isopropanol formation. When there is no growth, there is a high availability of ATP, which limits substrate uptake in order to avoid fueling the TCA cycle even further. Such behavior has a negative impact on product formation rates. To incentivize the cells to take up more acetate, the ATP surplus could be lowered by introducing an ATP-wasting mechanism [50, 51]. This could benefit isopropanol formation, but only if it is tightly controlled: if the resulting ATP turnover is too large, then the TCA cycle flux may increase withdrawing carbon from the product pathway. Hence, ATP wasting should be kept at levels where the resulting Thl:TCA flux ratio reaches the optimal value of 1.

NADPH availability is another target for metabolic engineering, as it has a major impact on the balance between acetone and isopropanol. Our data suggest that the cells produced sufficient NADPH but competing reactions (e.g., soluble transhydrogenase UdhA) might have decreased its availability for product formation. Consequently, limiting reactions competing for NADPH by introducing flux controls could enhance isopropanol formation. Using an NADH-dependent alcohol dehydrogenase in the pathway instead could have similar effects as providing more NADPH, because it would increase availability of the reducing agent (2 NADH per acetate entering the TCA cycle).

Finally, to shift cellular metabolism toward either growth or production, the Thl:TCA flux distribution could also be influenced by directly engineering either Thl itself or GltA. On glucose, the latter approach has already been investigated by designing a toggle switch for GltA [52, 53].

### Process design optimization

With our 2-phase process design, we achieved the highest reported isopropanol titers for *E. coli* cultivated on acetate and defined medium to date (Table 2). Likewise, we observed promising yields of 0.235 ± 0.030 mol mol^−1^ and acetone yield of 0.041 ± 0.006 mol mol^−1^ which constitute 67 % (isopropanol) and 79 % (isopropanol + acetone) of the energy-balanced maximum theoretical yield of 0.35 mol mol^−1^ of isopropanol per acetate, respectively. The yields obtained in this study are slightly lower compared to previous reports of 0.56 mol mol^−1^, which even exceeds the non-energy-balanced maximum theoretical yield of 0.50 mol mol^−1^. We attribute this discrepancy to the use of complex media additives like yeast extract in previous studies. Moving toward industrial-scale production, the use of such additives is economically not feasible. Nevertheless, for large-scale application further yield optimization is necessary.

**Table 2:** Comparison of isopropanol production on acetate with previous literature

Process strategy	Medium	Titer [g L^−1^]	Yield [mol mol^−1^]	Overall q_p_ [g g^−1^ h^−1^]	Source
Growth decoupled 25 °C, constitutive	Defined	4.85 ± 0.13	0.235 ± 0.030	0.053 ± 0.005	This study
Growth decoupled 25 °C, constitutive^a^	Defined	8.07 ± 0.13	0.166 ± 0.003	0.029 ± 0.002	This study
Growth decoupled 25 °C, constitutive^b^	Defined	13.3 ± 0.2	0.196 ± 0.004	0.023 ± 0.003	This study
Growth decoupled 37 °C, constitutive	Defined	5.90 ± 0.14	0.188 ± 0.005	0.081 ± 0.005	This study
Batch, inducible	Complex	1.44	0.56	n.a	[22]

^a^indicates ~ 6.5 g L^−1^ biomass for the N-starvation phase after 30 h of starvation

^b^indicates ~ 6.5 g L^−1^ biomass for the N-starvation phase after 115 h of starvation

Due to the inherent need for growth arrest while providing excess carbon, the N-starvation strategy remains a vital component for the process. Therefore, the focus for optimization lies on mitigating negative effects such as elevated stress levels, ensuring efficient carbon use and accelerating the overall process.

One way to achieve lower acetate stress levels is to implement an online acetate monitoring to optimize feeding. Ideally, acetate would still be available in excess, but its levels should be kept as low as possible to avoid toxicity.

Our data also suggest that lower temperatures are suitable measures against increased acetylation and excessive CO_2_-formation. However, low-temperature processes also experienced a significantly prolonged initial lag phase, thus lowering the space–time yield from 1.468 ± 0.063 mmol L^−1^ h^−1^ at 37 °C to 0.989 ± 0.114 mmol L^−1^ h^−1^ at 25 °C.

To overcome this challenge, a 2-stage continuous process design with a high-temperature growth reactor and a low-temperature N-starvation reactor could be implemented for efficient isopropanol production.

## Conclusions

In this study, we designed and constructed a constitutively expressed pathway for isopropanol formation and established a bioreactor production process on defined medium using acetate as carbon source. To establish knowledge on *E. coli* metabolism in such a scenario, we conducted an in-depth analysis of physiology and the proteome at two different temperatures while also modeling intracellular flux distributions.

Our process incorporated the need for a growth-decoupled design by implementing a nitrogen-starvation phase and allowed us to achieve the highest reported isopropanol titer on acetate to date (13.3 g L^−1^)–even with minimal medium without complex additives. Even though we observed only minor changes in abundance of product related enzymes between different conditions, we were able to confirm several stress conditions between different temperatures in response to N-starvation via proteome analysis. In future processes, mitigating acid stress, acetylation and improving cofactor availability in the N-starvation phase could help to increase cell viability. Examining intracellular flux distributions, we identified carbon precursor and especially ATP demand as the limiting factor for isopropanol production, which underlines carefully controlled low-demand conditions as most effective for efficient isopropanol formation. Moreover, characterization of the two general metabolic states of the growth-decoupled bioprocess can serve as a vantage point for improving metabolic and process engineering. Based on our findings, we assessed further optimization strategies to boost isopropanol production, which form a solid foundation for sustainable acetate upgrading and its large-scale implementation.

### Supplementary Information

Additional file 1. Additional information on strains, plasmids and primers, data of screening experiments, detailed proteomics results, additional modeling data.

Additional file 2. (Differentially) acetylated peptides at different temperatures grouped according to metabolic function.

Additional file 3. Comprehensive list of all investigated metabolic flux scenarios including all modeling input- and output data of the ECC2_comp model.

## Data Availability

For shotgun proteomics and acetylation proteomics, datasets including the raw data, search parameters as well identification/quantitation output was uploaded to the ProteomeXchange Consortium (http://proteomecentral.proteomexchange.org) via the PRIDE partner repository [35] with the dataset identifier PXD047939.
