# Supplementary material for: Efficiency of acetate-based isopropanol synthesis in Escherichia coli W is controlled by ATP demand
Source: Biotechnol Biofuels Bioprod. 2024 Aug 5;17:110. doi: 10.1186/s13068-024-02534-0 (PMC11302359; doi:10.1186/s13068-024-02534-0)
Supplement: Supplementary file 1 — Additional file 1. Additional information on strains, plasmids and primers, data of screening experiments, detailed proteomics results, additional modeling data. [file 13068_2024_2534_MOESM1_ESM.docx]

Additional material 1

Strains, plasmids and primers

**Table S1
List of strains used/created in this study.**

| strain | Source |
| --- | --- |
| *Escherichia coli* K-12 MG1655 DSM 18039 | DSMZ |
| *Escherichia coli* W DSM 1116 (=ATCC 9637) | DSMZ |
| *Escherichia coli* W *ΔldhA ΔadhE (E. coli* W KO2*)* | Kind gift of Prof. Michael Sauer (BOKU, Vienna, Austria) |
| *Escherichia coli* W *ΔldhA ΔadhE Δpta ΔfrdA (E. coli* W KO4*)* | Kind gift of Prof. Michael Sauer (BOKU, Vienna, Austria) |
| *Escherichia coli* TOP10^TM^ | Thermo Fisher Scientific (Thermo Fisher Scientific Inc. (NYSE: TMO)) |
| *Escherichia coli* TOP10^TM^ containing: |  |
| BB1_pIDTSmart(Kan^R^)_ FS2_*thl*_FS3 | This work |
| BB1_pIDTSmart(Kan^R^)_ FS2_atoDA_FS3 | This work |
| BB1_pIDTSmart(Kan^R^)_ FS2_adc_FS3 | This work |
| BB1_pIDTSmart(Kan^R^)_ FS2_*adh(NADPH)*_FS3 | This work |
| BB1_pIDTSmart(Kan^R^)_ FS2_*adh(NADH)*_FS3 | This work |
| BB2_pUC(Amp^R^)_ _LinkerA_p114_*thl*_TT_LinkerB | This work |
| BB2_pUC(Amp^R^)_ _LinkerB_p114_*atoDA*_TT_LinkerC | This work |
| BB2_pUC(Amp^R^)_ _LinkerC_p114_*adc*_TT_LinkerD | This work |
| BB2_pUC(Amp^R^)_ _LinkerD_p114_*adh(NADPH)*_TT_LinkerE | This work |
| BB2_pUC(Amp^R^)_ _LinkerD_p105_*adh(NADPH)*_TT_LinkerE | This work |
| BB2_pUC(Amp^R^)_ _LinkerD_p114_*adh(NADH)*_TT_LinkerE | This work |
| BB2_pUC(Amp^R^)_ _LinkerD_p105_*adh(NADH)*_TT_LinkerE | This work |
| IPA_P_p114 | This work |
| IPA_P_p105 | This work |
| IPA_H_p114 | This work |
| IPA_H_p105 | This work |
| *Escherichia coli* W DSM 1116 containing: |  |
| IPA_P_p114 | This work |
| IPA_P_p105 | This work |
| IPA_H_p114 | This work |
| IPA_H_p105 | This work |
| *Escherichia coli* W *ΔldhA ΔadhE* containing: |  |
| IPA_P_p114 | This work |
| IPA_P_p105 | This work |
| IPA_H_p114 | This work |
| IPA_H_p105 | This work |
| *Escherichia coli* W *ΔldhA ΔadhE Δpta ΔfrdA* containing: |  |
| IPA_P_p114 | This work |
| IPA_P_p105 | This work |
| IPA_H_p114 | This work |
| IPA_H_p105 | This work |

**Table S2
List of plasmids used/created in this work.**

| Name | ID | Source |
| --- | --- | --- |
| BB1_pIDTSmart(Kan^R^) |  |  |
| _FS1_p105_FS2 |  | (Sarkari et al., 2017) |
| _FS1_p114_FS2 |  | (Sarkari et al., 2017) |
| _FS3_TT_FS4 |  | (Sarkari et al., 2017) |
| _FS2_amilCP_FS3 |  | (Sarkari et al., 2017) |
| _FS2_thl_FS3 |  | this work |
| _FS2_atoDA_FS3 |  | this work |
| _FS2_adc_FS3 |  | this work |
| _FS2_adh(NADPH)_FS3 |  | this work |
| _FS2_adh(NADH)_FS3 |  | this work |
| BB2_pUC(Amp^R^) |  |  |
| _LinkerA_FS1_FS4_LinkerB |  | (Sarkari et al., 2017) |
| _LinkerB_FS1_FS4_LinkerC |  | (Sarkari et al., 2017) |
| _LinkerC_FS1_FS4_LinkerD |  | (Sarkari et al., 2017) |
| _LinkerD_FS1_FS4_LinkerE |  | (Sarkari et al., 2017) |
| _LinkerA_p114_thl_TT_LinkerB |  | this work |
| _LinkerB_p114_atoDA_TT_LinkerC |  | this work |
| _LinkerC_p114_adc_TT_LinkerD |  | this work |
| _LinkerD_p114_adh(NADPH)_TT_LinkerE |  | this work |
| _LinkerD_p105_adh(NADPH)_TT_LinkerE |  | this work |
| _LinkerD_p114_adh(NADH)_TT_LinkerE |  | this work |
| _LinkerD_p105_adh(NADH)_TT_LinkerE |  | this work |
| BB3_pUC(Kan^R^) |  |  |
| _LinkerAE |  | (Sarkari et al., 2017) |
| _LinkerA_p114_thl_p114_atoDA_p114_adc_p114_adh(NADPH)_LinkerE | IPA_P_p114 | this work |
| _LinkerA_p114_thl_p114_atoDA_p114_adc_p105_adh(NADPH)_LinkerE | IPA_P_p105 | this work |
| _LinkerA_p114_thl_p114_atoDA_p114_adc_p114_adh(NADH)_LinkerE | IPA_H_p114 | this work |
| _LinkerA_p114_thl_p114_atoDA_p114_adc_p105_adh(NADH)_LinkerE | IPA_H_p105 | this work |

**Table S3
List of primers used for sequencing and PCR amplification of atoDA.**

| atoDA forward | 5’ –GATCGGTCTCAAAGCTCATAAATCACCCCGTTG– 3’ |
| --- | --- |
| atoDA reverse | 5’ – GATCGGTCTCACATGAAAACAAAATTGATGACATTACAAG – 3’ |
| Seq_forward | 5’ – GCAGTCCAGTTACGCTG – 3’ |
| Seq reverse | 5’ – CGTGGACCGATCATACG – 3’ |


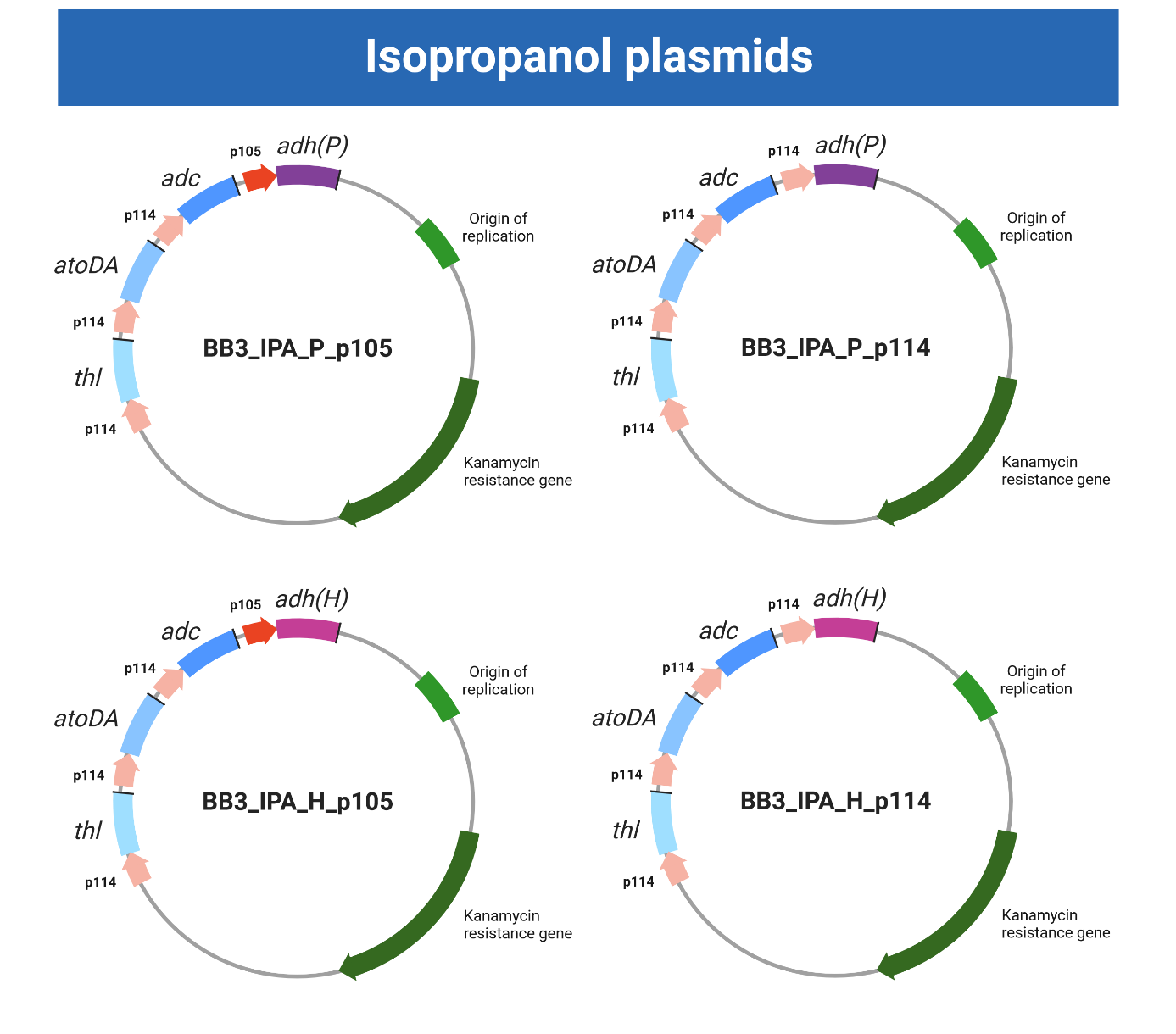


**Fig. S1.** Plasmids with different versions of the isopropanol production pathway. Promoter (orange/red arrow; BBa_ J23114 from Anderson promoter library = p114; BBa_ J23105 from Anderson promoter library = p105; p105 is about 2.5 times stronger than p114) and terminator (black line) were placed around every gene for improved expression control. H = construct containing NADH-dependent adh1 from Gordonia sp. TY-5, P = construct containing NADPH-dependent adh from C. beijerinckii. Created with BioRender.com

Codon optimized sequences of the pathway genes

Thl – acetyl-coA acetyltransferase thiolase – *Clostridium acetobutylicum* ATCC824 / DSM792

5‘ -ATGAAAGAGGTAGTCATTGCGAGTGCGGTGCGTACCGCTATCGGCTCCTACGGTAAATCGCTGAAGGATGTTCCGGCGGTCGACCTGGGCGCCACGGCTATTAAGGAAGCCGTCAAAAAGGCTGGCATCAAACCTGAAGATGTGAATGAAGTTATTTTAGGTAATGTCCTGCAGGCTGGCCTGGGTCAAAACCCCGCACGCCAGGCCAGTTTCAAAGCGGGTCTGCCAGTTGAGATCCCGGCCATGACCATCAACAAAGTTTGTGGTAGCGGTCTGCGCACAGTGTCACTGGCGGCACAGATCATCAAAGCGGGCGATGCCGACGTGATCATTGCGGGTGGTATGGAGAACATGTCGCGCGCACCATACCTGGCTAATAACGCGCGTTGGGGTTACCGTATGGGGAACGCGAAATTCGTGGATGAAATGATCACGGACGGCCTCTGGGATGCCTTTAACGACTACCACATGGGCATTACTGCGGAAAATATCGCGGAACGTTGGAATATTTCACGCGAAGAGCAGGACGAGTTCGCCCTCGCATCCCAAAAAAAAGCAGAGGAAGCTATCAAATCCGGTCAGTTTAAGGATGAAATCGTGCCCGTTGTGATTAAAGGTCGGAAGGGTGAAACAGTTGTTGATACCGATGAACACCCCCGCTTCGGTTCTACCATCGAAGGGTTGGCGAAACTGAAACCGGCGTTTAAAAAAGACGGCACCGTTACGGCTGGCAACGCTAGCGGACTGAATGATTGCGCGGCGGTTCTGGTGATTATGTCGGCTGAAAAAGCCAAAGAACTCGGCGTGAAGCCGCTGGCTAAAATCGTATCCTACGGTTCGGCCGGCGTTGATCCGGCGATTATGGGTTACGGGCCATTCTATGCGACCAAAGCAGCTATTGAAAAAGCGGGGTGGACCGTAGATGAACTGGATTTGATCGAATCAAACGAAGCGTTCGCGGCTCAAAGCCTGGCTGTCGCTAAGGACTTGAAATTTGACATGAACAAAGTTAACGTCAATGGTGGGGCTATCGCACTGGGCCATCCTATCGGCGCAAGCGGTGCGCGTATTCTGGTTACCCTGGTACATGCGATGCAGAAACGCGATGCTAAAAAAGGTCTGGCGACACTGTGCATCGGCGGGGGGCAAGGTACCGCGATTTTGTTAGAAAAATGTTAA-3‘

Adc – acetoacetate decarboxylase – *Clostridium acetobutylicum* ATCC824 / DSM792

5’ -ATGTTAAAAGATGAAGTTATCAAGCAGATCTCGACTCCACTTACGAGCCCGGCATTTCCGCGCGGCCCATACAAATTTCATAATCGCGAATATTTTAATATCGTGTACCGTACAGACATGGATGCTCTGCGCAAAGTGGTACCGGAACCTCTTGAGATCGATGAGCCGTTGGTACGTTTTGAAATTATGGCGATGCATGATACTTCGGGTTTAGGATGTTATACCGAATCTGGACAAGCGATTCCTGTATCTTTCAATGGGGTCAAAGGTGATTACCTGCATATGATGTATCTGGATAATGAACCGGCAATTGCAGTCGGACGCGAGCTGTCAGCTTACCCCAAAAAACTGGGTTATCCAAAACTGTTCGTGGATTCCGATACGCTTGTCGGGACCTTAGATTATGGTAAGCTGCGCGTTGCGACCGCGACCATGGGCTACAAACACAAAGCTCTGGACGCTAACGAGGCGAAAGACCAGATCTGTCGCCCGAACTACATGCTGAAGATCATTCCTAATTATGATGGCAGCCCTCGTATTTGCGAATTGATTAATGCTAAAATTACTGATGTAACCGTGCACGAGGCTTGGACCGGCCCGACTCGCCTTCAACTGTTTGATCATGCAATGGCGCCGCTGAATGATCTGCCGGTTAAAGAAATTGTGTCGTCATCACACATTTTGGCGGATATTATCCTTCCGCGTGCTGAAGTTATTTATGACTATCTGAAATAA-3’

Adh NADP-dependent secondary alcohol dehydrogenase – *Clostridium beijerinckii*

5‘-ATGAAAGGCTTTGCAATGCTGGGTATCAATAAACTGGGTTGGATTGAAAAAGAACGTCCGGTGGCGGGGTCTTACGACGCGATTGTACGCCCGCTGGCGGTTAGCCCATGCACCAGTGATATTCACACGGTGTTTGAAGGCGCACTGGGTGATCGCAAAAACATGATTCTTGGTCATGAGGCTGTTGGGGAAGTAGTGGAAGTCGGCTCAGAAGTTAAAGATTTCAAACCGGGCGACCGGGTGATCGTACCGTGCACCACGCCAGACTGGCGTTCTCTGGAAGTGCAGGCGGGGTTTCAGCAGCACAGCAACGGTATGCTGGCAGGCTGGAAATTCTCCAATTTTAAAGACGGAGTGTTTGGGGAATATTTCCACGTTAATGATGCCGACATGAACCTGGCTATCCTGCCTAAAGACATGCCGCTGGAAAACGCTGTTATGATCACGGATATGATGACAACGGGCTTTCACGGGGCAGAGCTCGCGGATATTCAGATGGGCTCGTCCGTGGTAGTAATCGGGATTGGTGCAGTGGGCCTGATGGGTATCGCGGGCGCGAAGCTTCGGGGGGCCGGCCGCATTATTGGGGTGGGCAGCCGCCCGATTTGCGTGGAAGCGGCCAAATTCTATGGTGCGACGGATATTCTGAATTACAAAAATGGTCACATCGTGGATCAGGTGATGAAGTTAACAAATGGGAAAGGCGTGGATCGGGTGATTATGGCGGGCGGAGGCAGCGAAACGTTGAGCCAAGCGGTATCGATGGTCAAACCAGGCGGTATCATCAGCAACATCAATTATCATGGCTCAGGTGACGCGCTCTTAATTCCGCGCGTTGAATGGGGTTGCGGAATGGCCCACAAAACGATCAAGGGTGGTCTGTGCCCGGGCGGCCGTTTACGCGCCGAAATGCTGCGTGATATGGTTGTTTACAACCGCGTAGATCTGTCAAAACTGGTAACTCATGTGTACCATGGGTTTGACCATATCGAGGAAGCACTGCTGCTGATGAAAGATAAACCTAAGGACCTGATTAAAGCCGTGGTCATTCTGTAA-3‘

Adh – NADH-dependent secondary alcohol dehydrogenase – *Gordonia sp.* TY-5

5’-ATGCGCGCCGTACAGGTAGTGGGCTACCATGATAAATTACAGTTGAACGAAATTCCTGATCCTGAGATTACCGGTCCGTTGGATGTTATTGTGCGTATCGGCGGAGCAGGCGTCTGCCGTACGGATCTGCATATTCTGGAAGGCCAATGGGAAGCTAAAAGCGGCGTTGCCCTGCCGTACACGATTGGCCATGAAAACGCCGGGTGGGTAGAAGCCATTGGGGATGCCGTGACCAATGTTGCGGTGGGTGATAAAGTTATTCTGCACCCACTGATCACCTGCGGTCTGTGCCGCGCGTGTCGCGATGGGGATGATGTGCATTGTGAGAACAGCAAATTCCCGGGCATCGATACCGATGGTGGGTACGCCGATTATTTAAAAACTACCGCACGGAGCGTGGTACGCATTGATGATAGCCTGGAACCAGCCGCGGTAGCTGCGCTTGCCGATGCCGGACTGACTGCTTACCATGCCGCGGCAAAAGTTGCGCGCATGACACGCCCAGGCGATACCTGCGTTGTGATTGGCGCTGGCGGTTTAGGGCACATTGGAATCCAAGTGTTGAGCGCAATTTCTGGTGTTACCATTGTGGTACTGGATCGCAATCCGGACGCCGTCGCGCTGGCTGTCGAAGTGGGTGCGCATGAAGGAATCGTGGCCGATGGTAACCATATTGAAAAGGTTCTGGCGCTGACGGATGGCAAAGGCGCAGAAGCAGTGTTAGATTTTGTGGGCGAAGGTGGGGCAACTGCGGAAGGTGTCGCGATGCTCCGTCAAGCGGGGAACTATTTCGTTGTAGGGTACGGAGAGAATATCAATGTGCCGACCATCGATATTATCTCCACTGAGATTAACTTTATCGGTAACCTCGTTGGCTCCTACAACGATTTGACTGAACTCATGACCCTGGCAGCACAGGGTAAAGTTACGCTGCATACGGCGACGTATCCCCTTACCGACTTCCAGCAGGCACTGGACGACCTGGACGCGGGCAAAGTGCGTGGCCGTGCCATTCTGATTCCGTAA-3’

Shake flask experiments

| 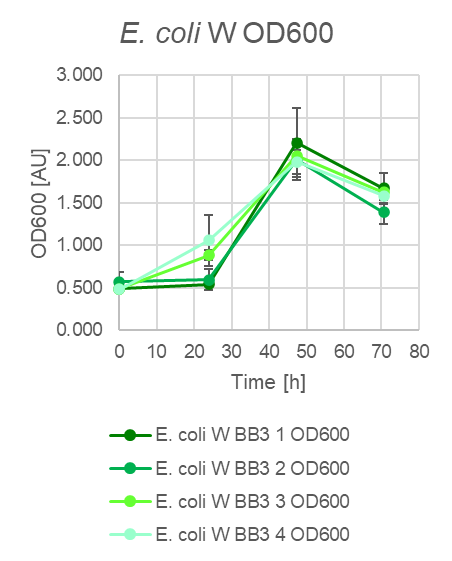  **a)** | 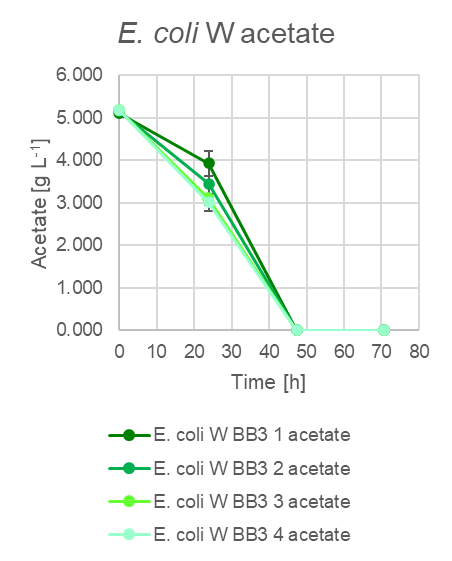  **b)** |
| --- | --- |
| 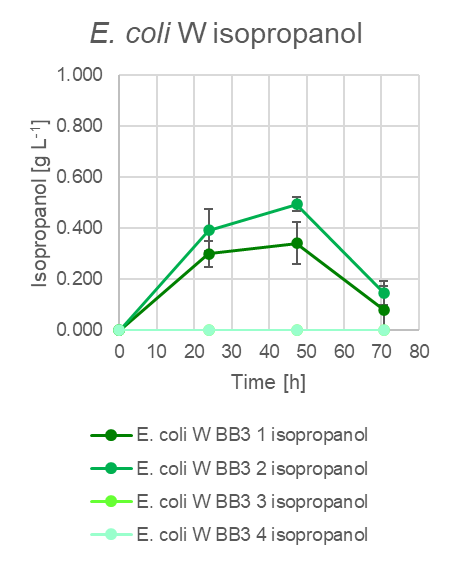  **c)** | 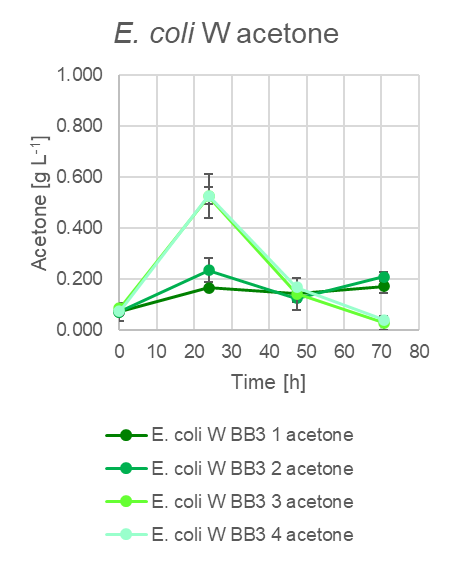  **d)** |

**Fig. S2.** Concentration profiles of E. coli W biological triplicates of screened strains in shake flasks at 25 °C and 230 rpm on 5 g L^-1^ acetate. Error bars denote the standard deviations of the means. a) Optical density at 600 nm. b) Acetate concentration. c) Isopropanol concentration. d) Acetone concentration. BB3 1 = IPA_P_p114. BB3 2 = IPA_P_p105. BB3 3 = IPA_H_p114. BB3 4 = IPA_H_p105.

| 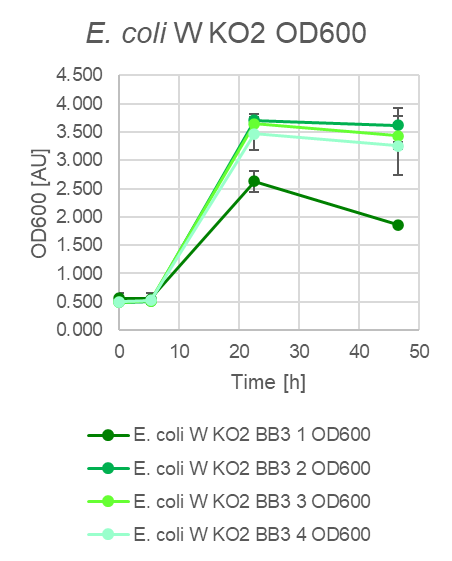  **a)** | 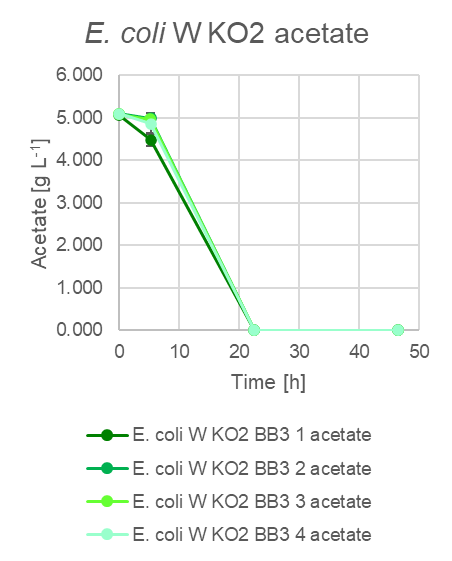  **b)** |
| --- | --- |
| 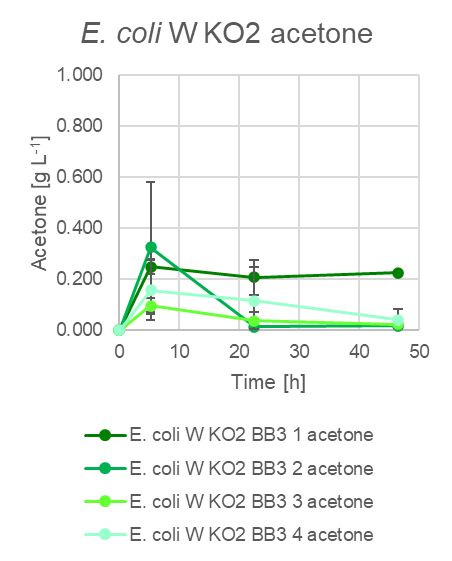  **c)** | 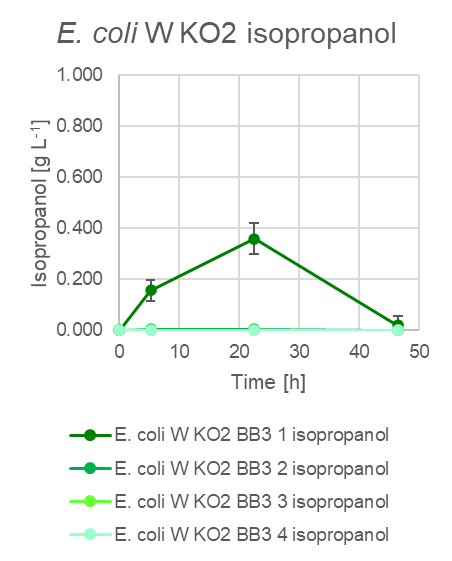  **d)** |

**Fig. S3.** Concentration profiles of E. coli W KO2 biological triplicates of screened strains in shake flasks at 25 °C and 230 rpm on 5 g L^-1^ acetate. Error bars denote the standard deviations of the means. a) Optical density at 600 nm. b) Acetate concentration. c) Acetone concentration. d) Isopropanol concentration. BB3 1 = IPA_P_p114. BB3 2 = IPA_P_p105. BB3 3 = IPA_H_p114. BB3 4 = IPA_H_p105.

| 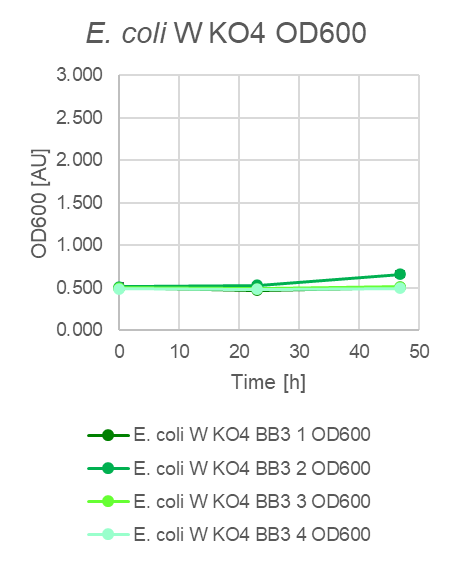  **a)** | 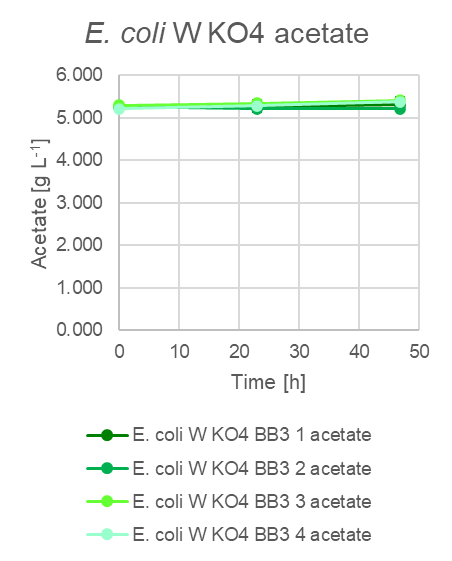  **b)** |
| --- | --- |
| 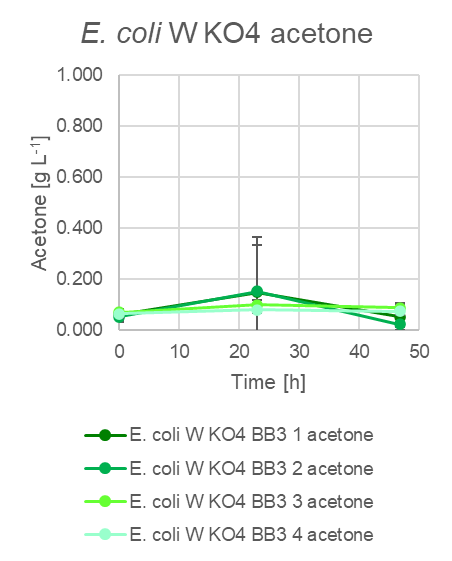  **c)** | 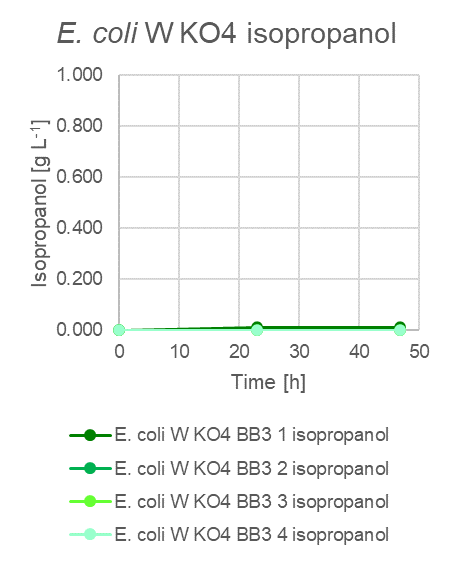  **d)** |

**Fig. S4.** Concentration profiles of E. coli W KO4 biological triplicates of screened strains in shake flasks at 25 °C and 230 rpm on 5 g L^-1^ acetate. Error bars denote the standard deviation of the means. a) Optical density at 600 nm. b) Acetate concentration. c) Acetone concentration. d) Isopropanol concentration. BB3 1 = IPA_P_p114. BB3 2 = IPA_P_p105. BB3 3 = IPA_H_p114. BB3 4 = IPA_H_p105.

Complete proteomics results


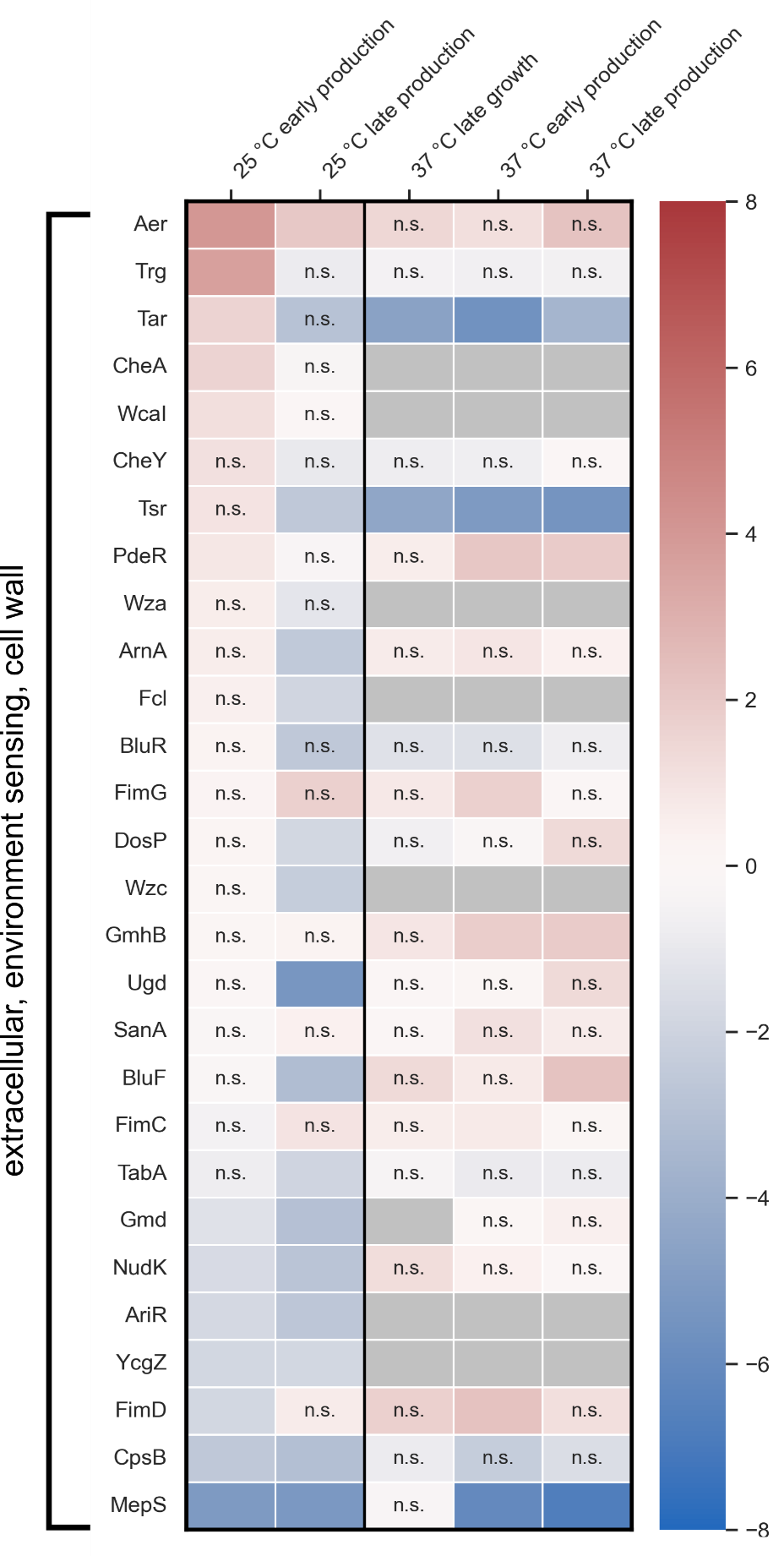


**Fig. S5.** Complete within-temperature log2 fold changes of proteins associated with extracellular processes, environment sensing or cell wall synthesis relative to the first -omics sample of the respective temperature. “n.s.” (“not significant”) indicates a non-significant fold change. Gray tiles denote that the protein was not detected in the sample.


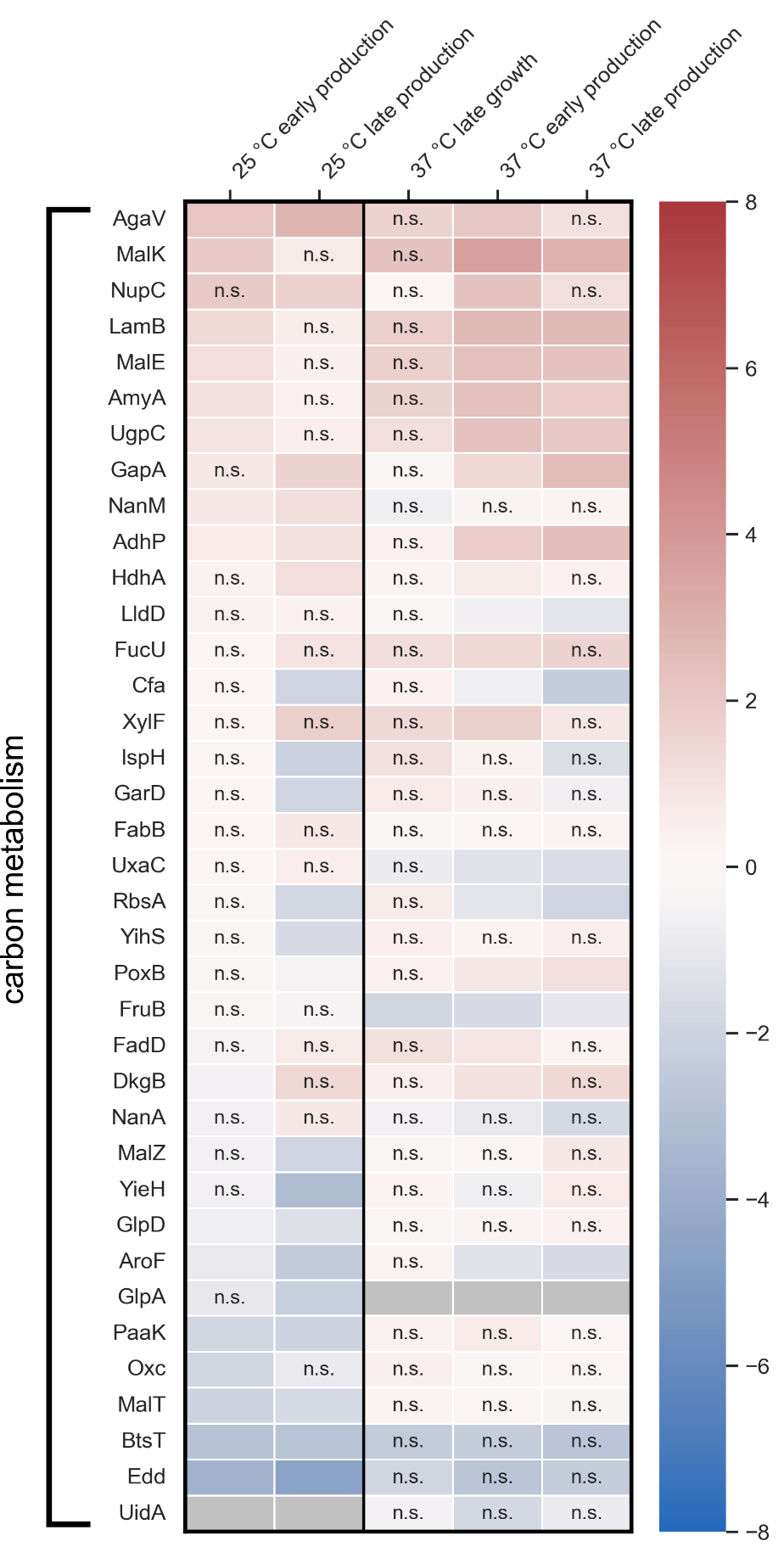


**Fig. S6.** Complete within-temperature log2 fold changes of proteins associated with carbon metabolism relative to the first -omics sample of the respective temperature. “n.s.” (“not significant”) indicates a non-significant fold change. Gray tiles denote that the protein was not detected in the sample.


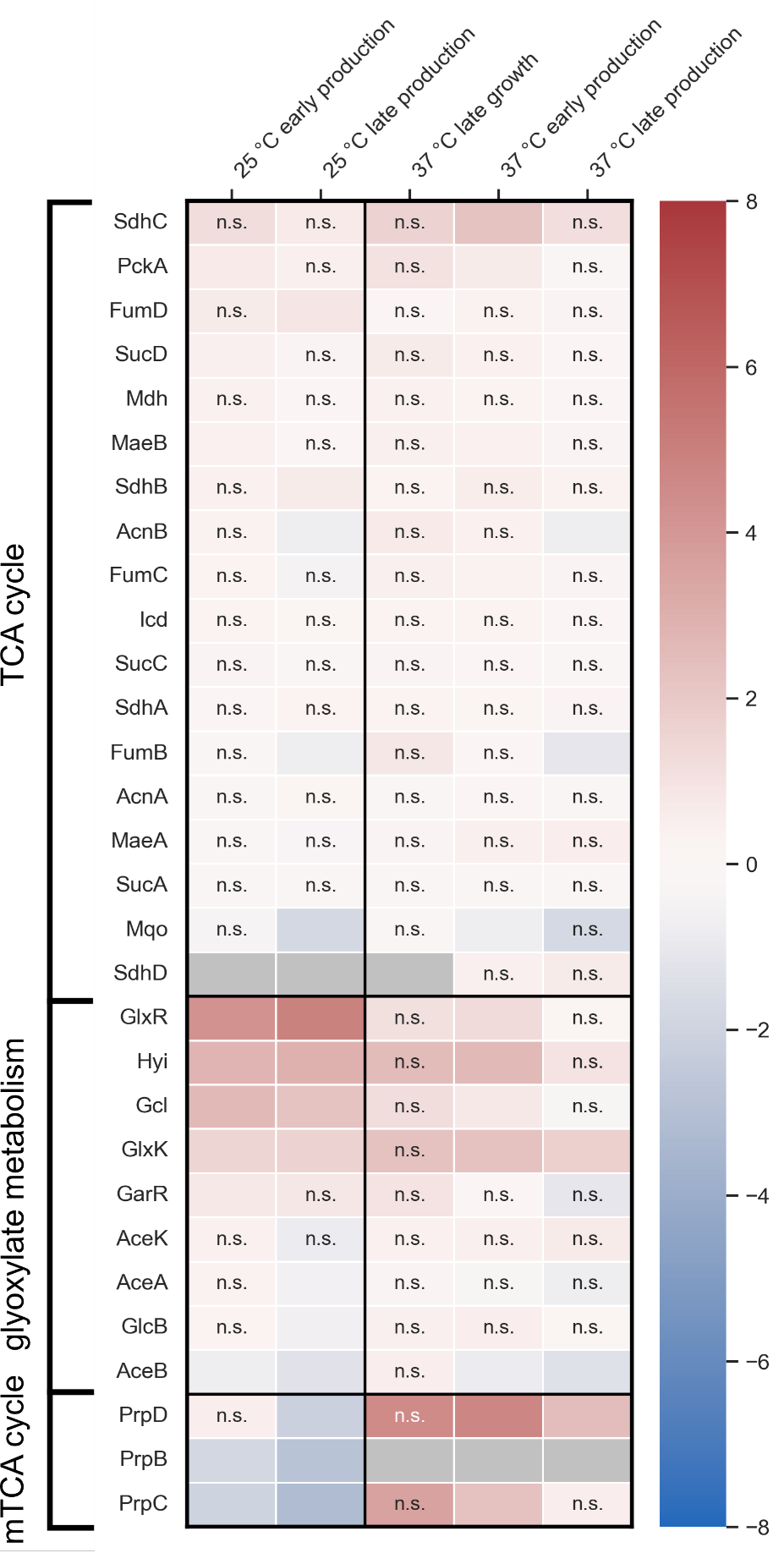


**Fig. S7.** Complete within-temperature log2 fold changes of proteins associated with TCA cycle, mTCA cycle and glyoxylate metabolism relative to the first -omics sample of the respective temperature. “n.s.” (“not significant”) indicates a non-significant fold change. Gray tiles denote that the protein was not detected in the sample.


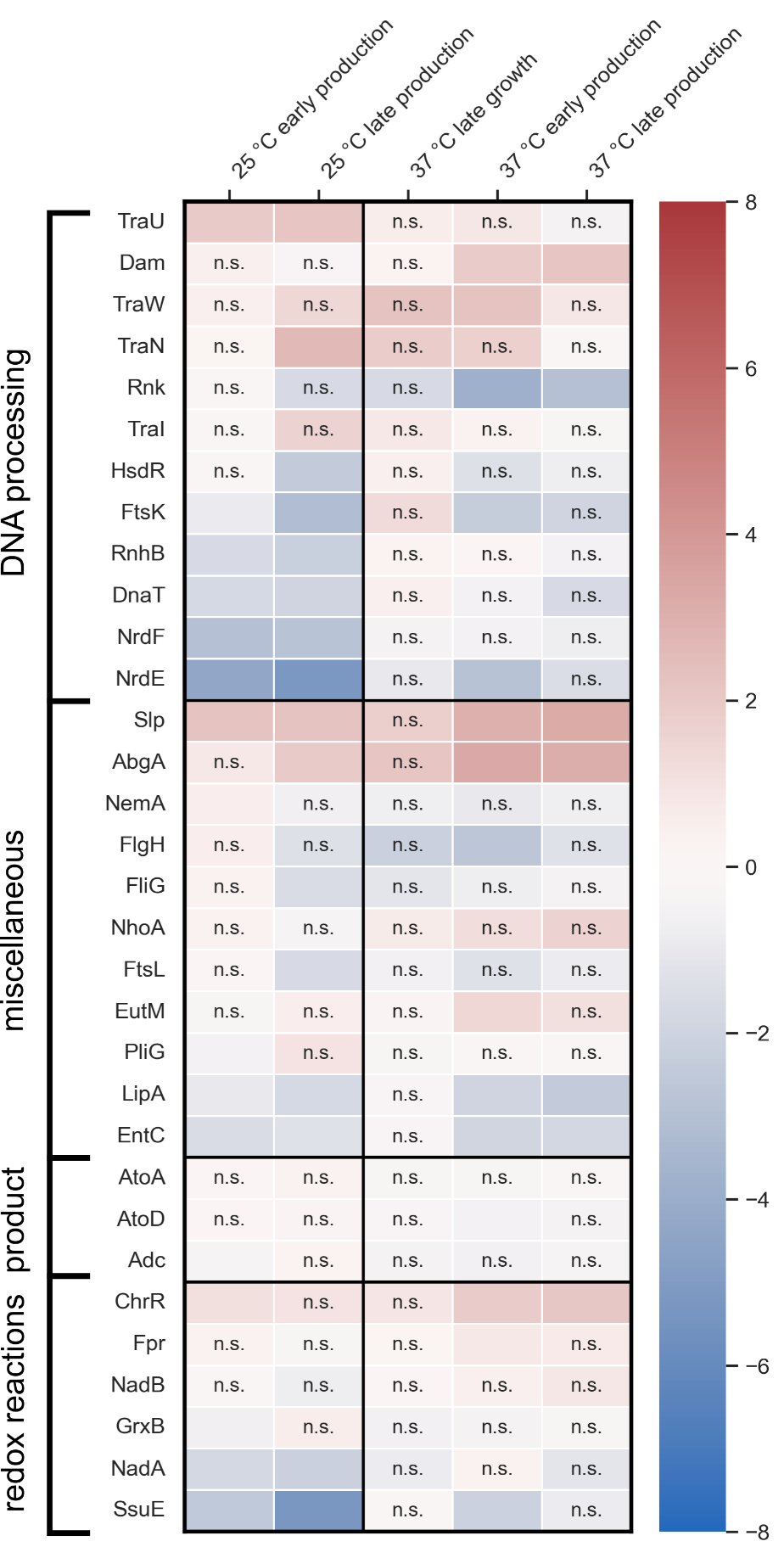


**Fig. S8.** Complete within-temperature log2 fold changes of proteins associated with DNA processing, product pathway, redox reactions and miscellaneous relative to the first -omics sample of the respective temperature. “n.s.” (“not significant”) indicates a non-significant fold change. Gray tiles denote that the protein was not detected in the sample.


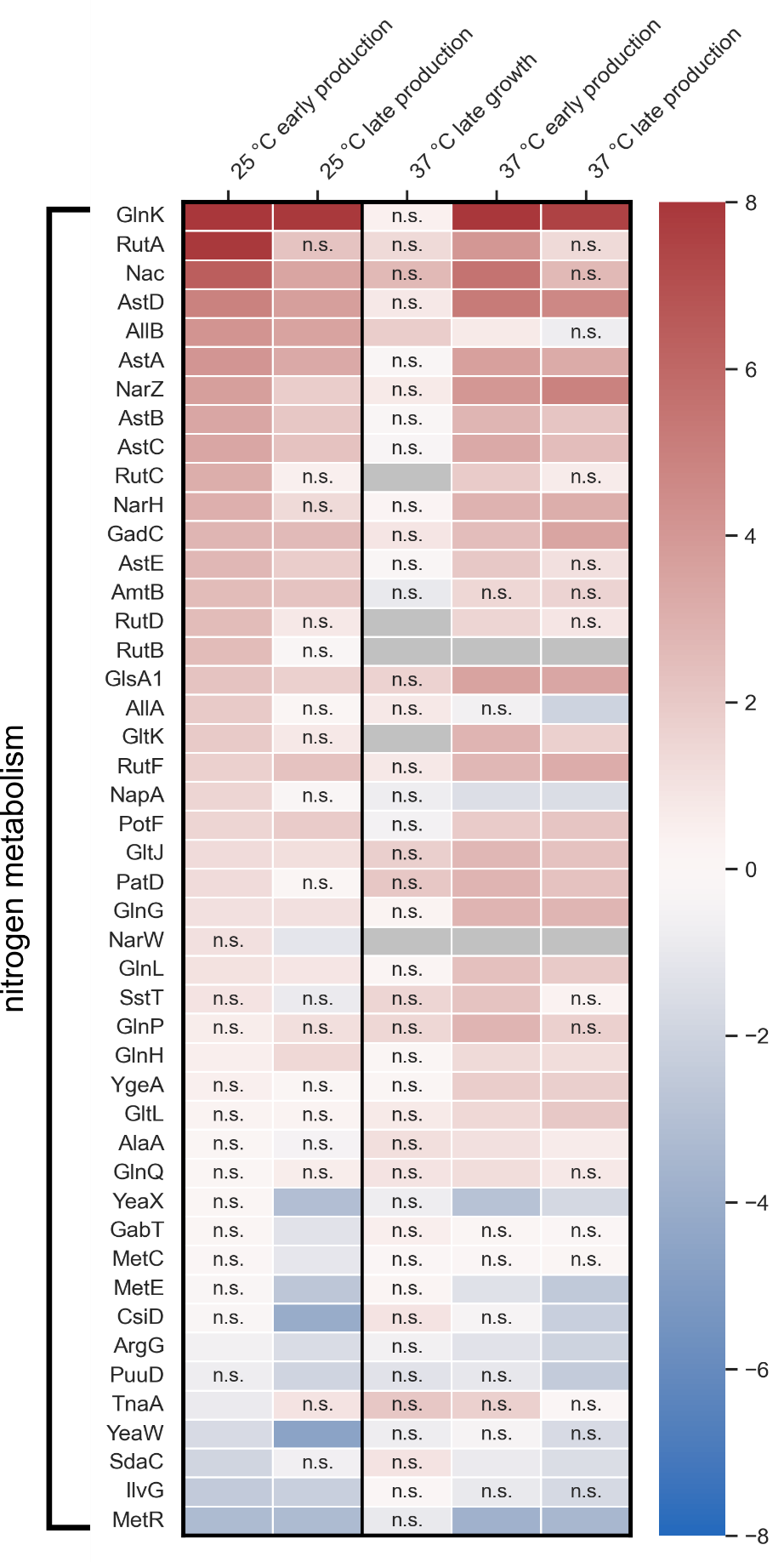


**Fig. S9.** Complete within-temperature log2 fold changes of proteins associated with nitrogen metabolism relative to the first -omics sample of the respective temperature. “n.s.” (“not significant”) indicates a non-significant fold change. Gray tiles denote that the protein was not detected in the sample.


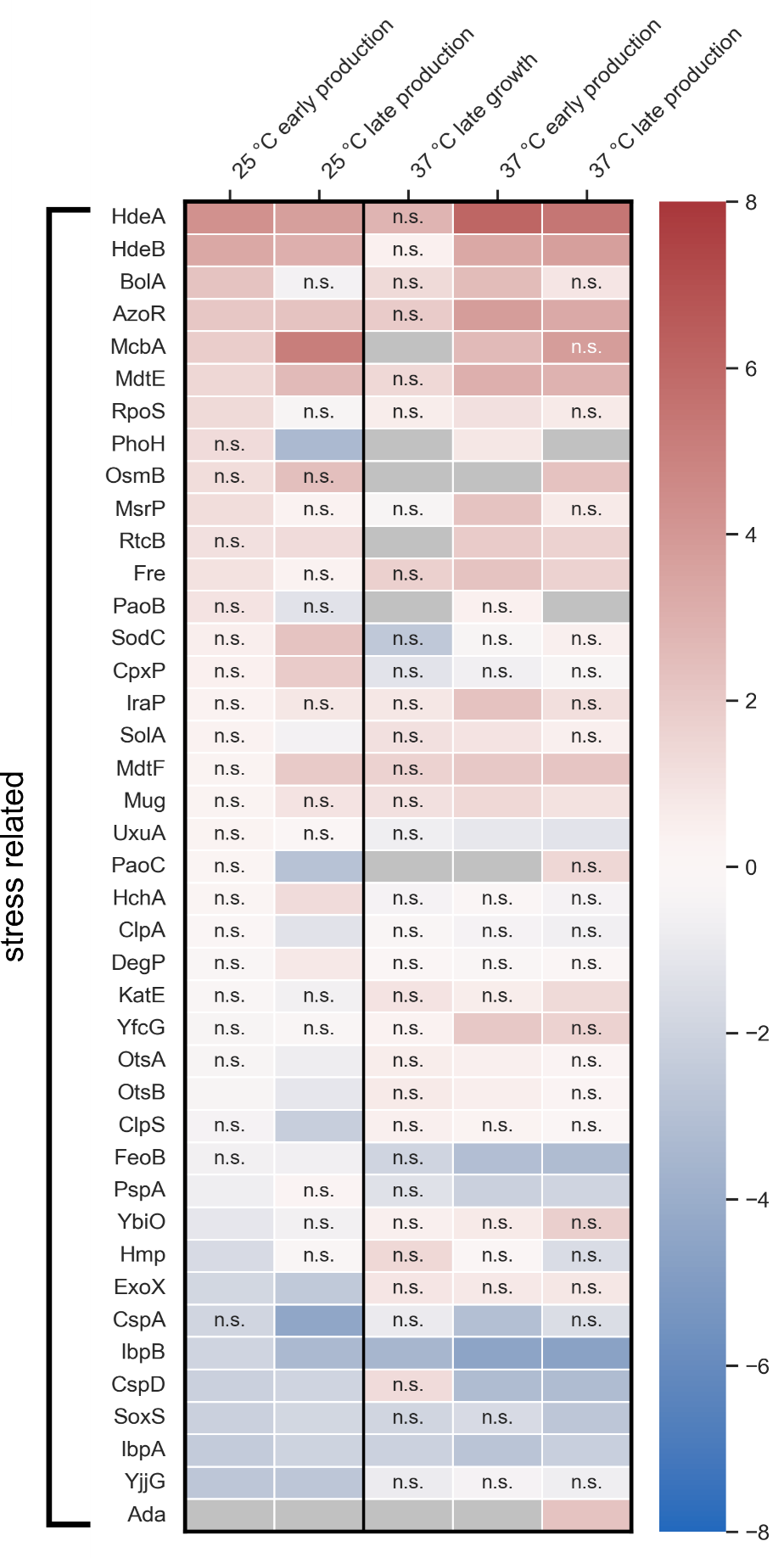


**Fig. S10.** Complete within-temperature log2 fold changes of proteins associated with stress relative to the first -omics sample of the respective temperature. “n.s.” (“not significant”) indicates a non-significant fold change. Gray tiles denote that the protein was not detected in the sample.


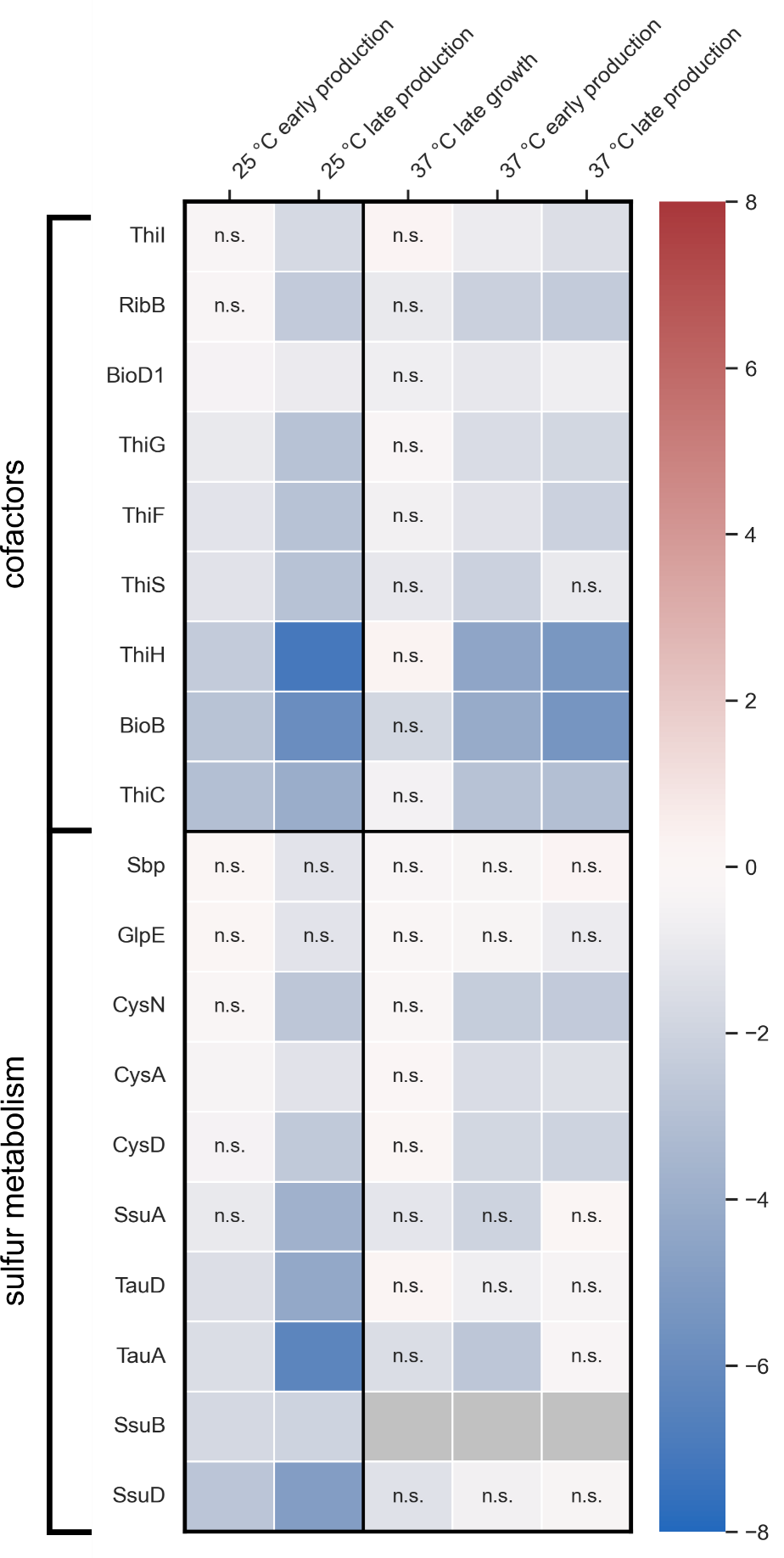


**Fig. S11.** Complete within-temperature log2 fold changes of proteins associated with cofactor formation and sulfur metabolism relative to the first -omics sample of the respective temperature. “n.s.” (“not significant”) indicates a non-significant fold change. Gray tiles denote that the protein was not detected in the sample.


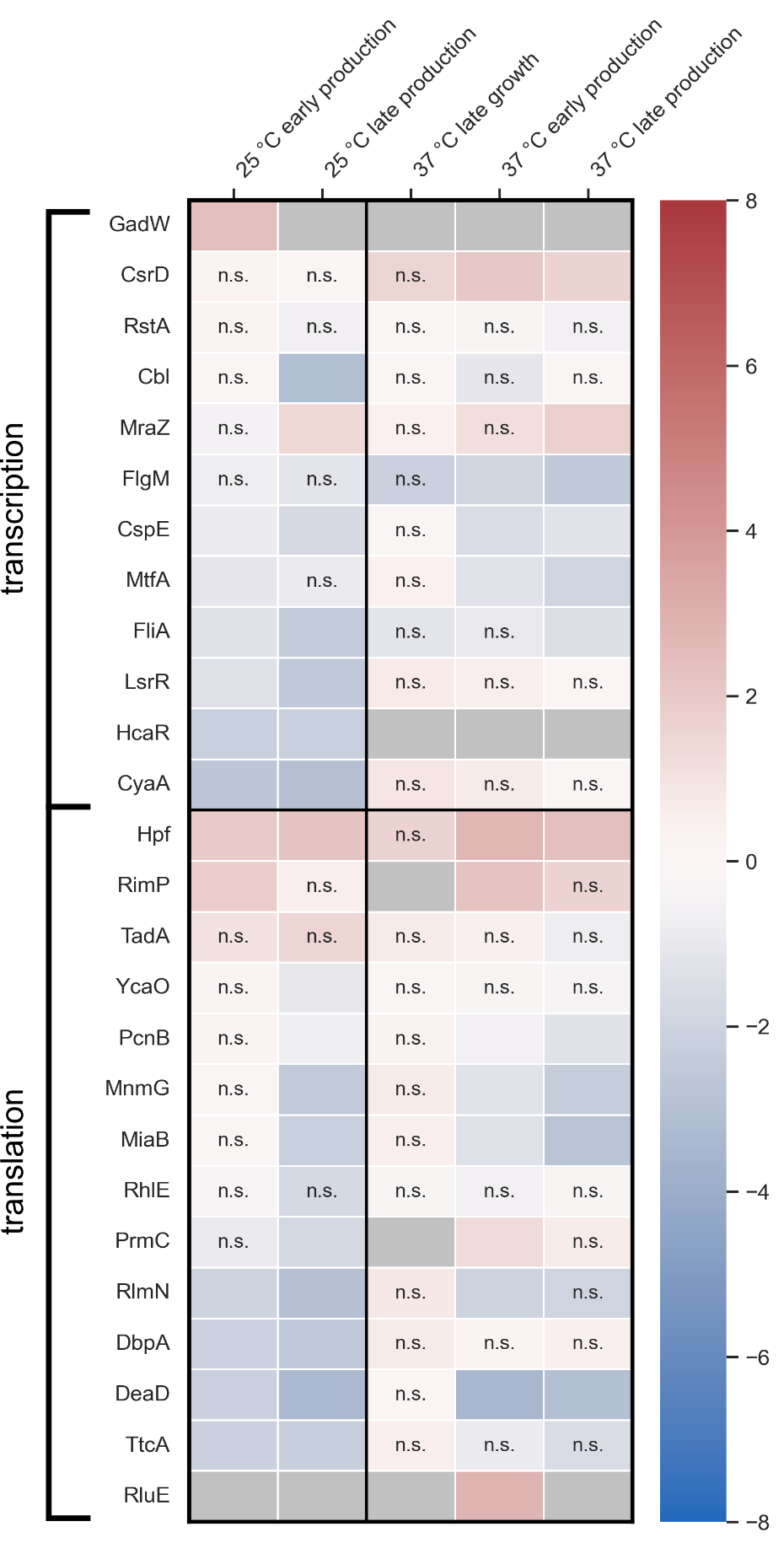


**Fig. S12.** Complete within-temperature log2 fold changes of proteins associated with transcription and translation relative to the first -omics sample of the respective temperature. “n.s.” (“not significant”) indicates a non-significant fold change. Gray tiles denote that the protein was not detected in the sample.


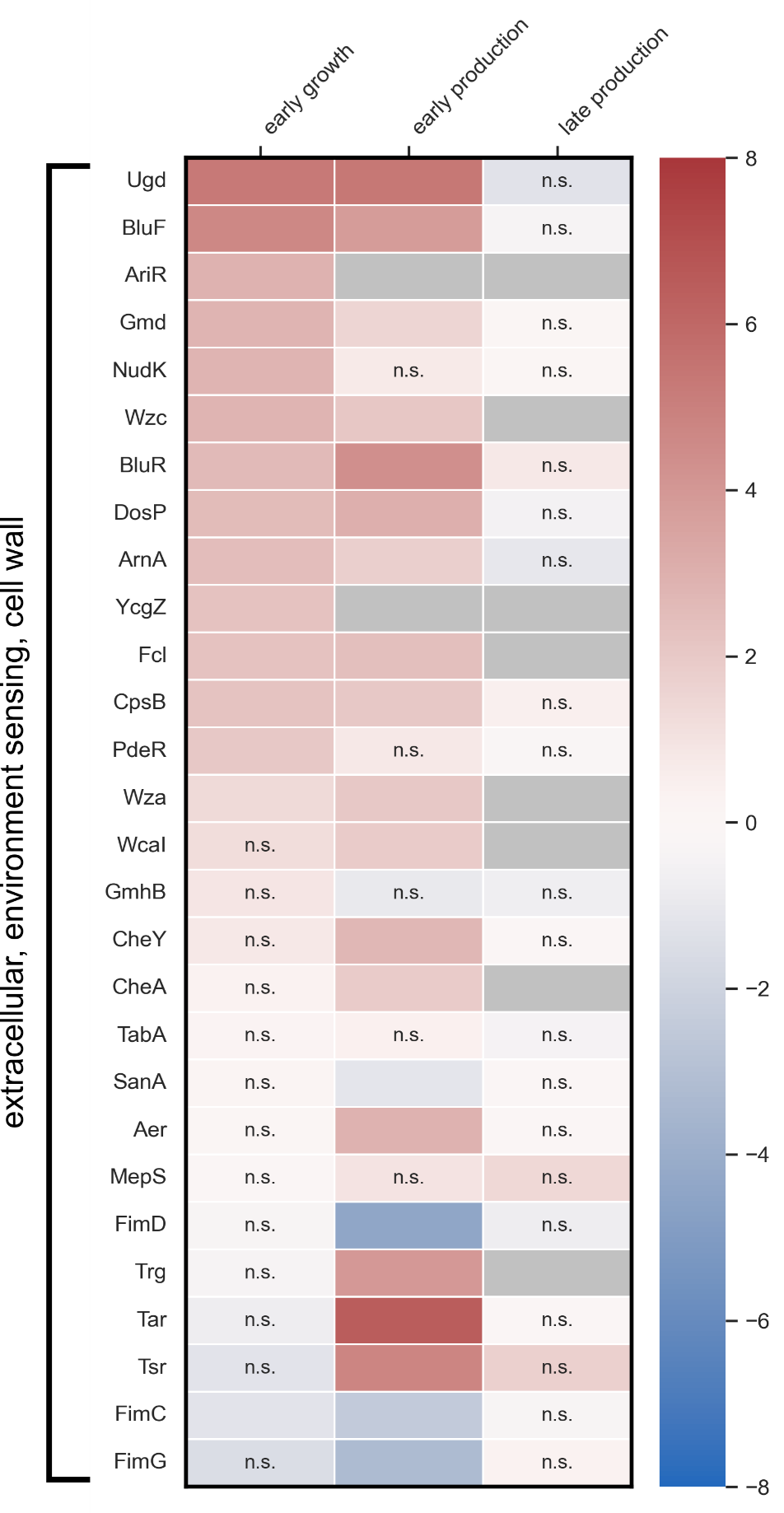


**Fig. S13.** Complete across-temperature log2 fold changes of proteins associated with extracellular processes, environment sensing and cell wall formation. For every compared phase between the different temperature processes the 25 °C sample was used as a reference (therefore red and blue color indicates a higher abundance at 25 °C and 37 °C respectively). “n.s.” (“not significant”) indicates a non-significant fold change. Gray tiles denote that the protein was not detected in the sample.


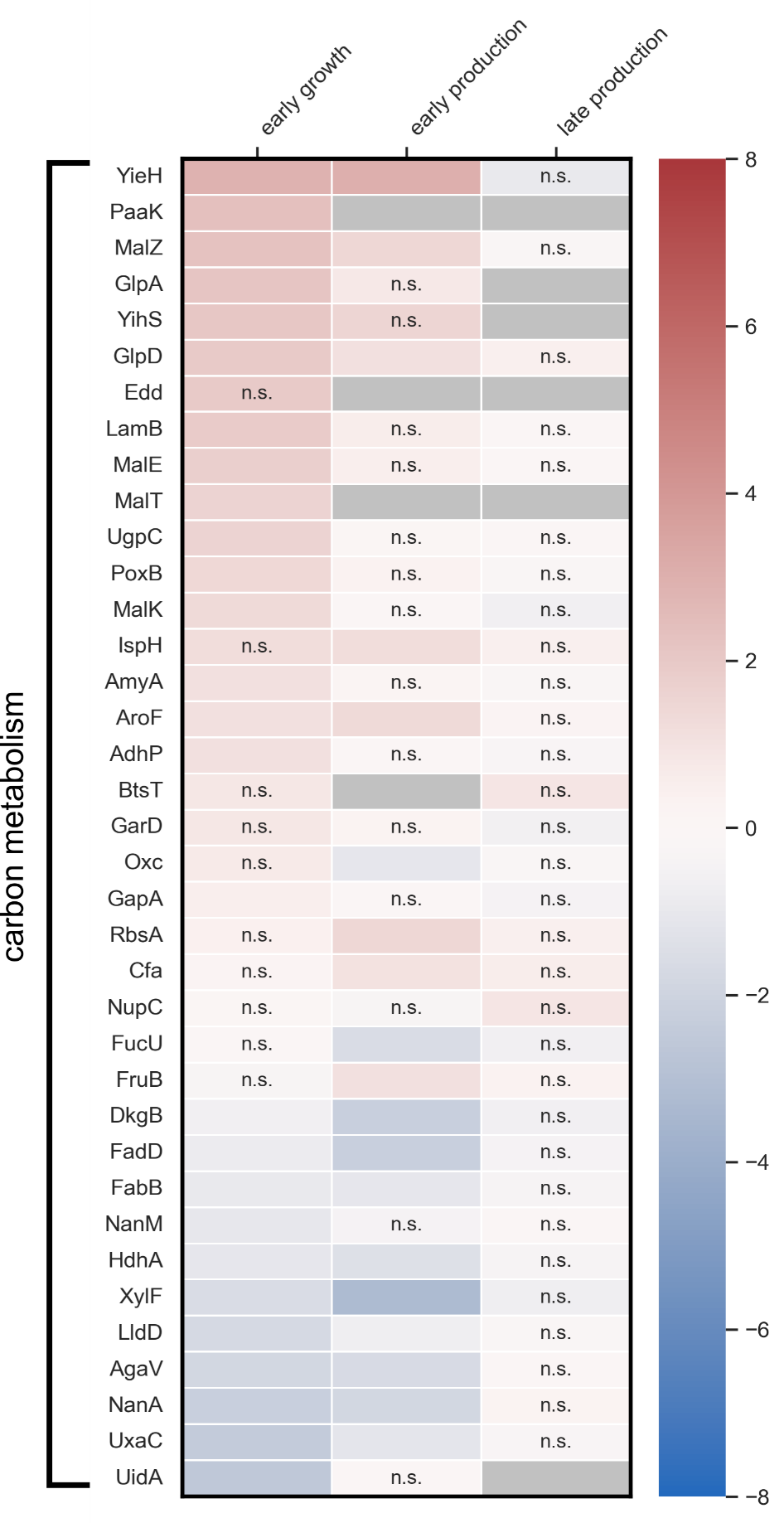


**Fig. S14.** Complete across-temperature log2 fold changes of proteins associated with carbon metabolism. For every compared phase between the different temperature processes the 25 °C sample was used as a reference (therefore red and blue color indicates a higher abundance at 25 °C and 37 °C respectively). “n.s.” (“not significant”) indicates a non-significant fold change. Gray tiles denote that the protein was not detected in the sample.


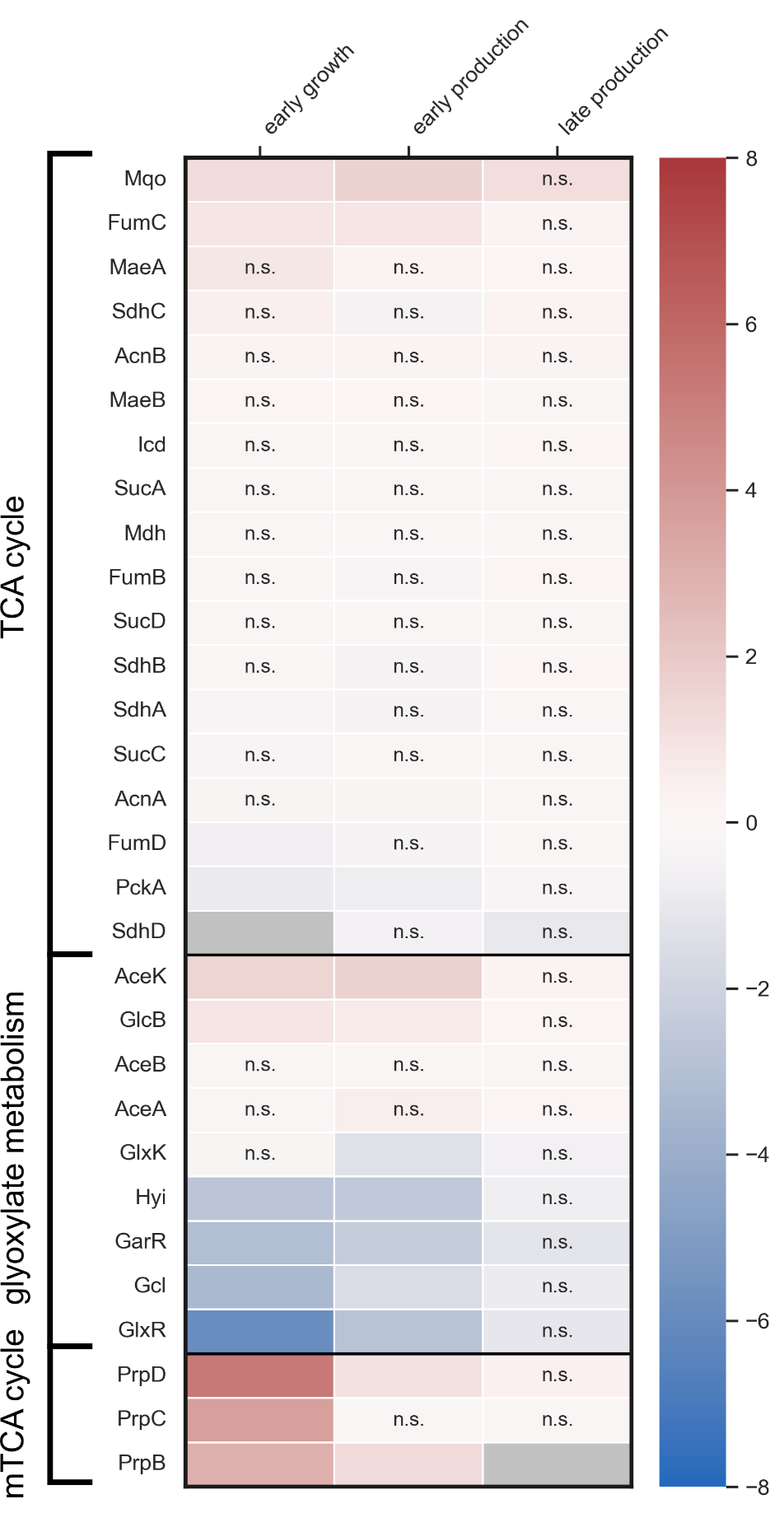


**Fig. S15.** Complete across-temperature log2 fold changes of proteins associated with TCA cycle, mTCA cycle and glyoxylate metabolism. For every compared phase between the different temperature processes the 25 °C sample was used as a reference (therefore red and blue color indicates a higher abundance at 25 °C and 37 °C respectively). “n.s.” (“not significant”) indicates a non-significant fold change. Gray tiles denote that the protein was not detected in the sample.


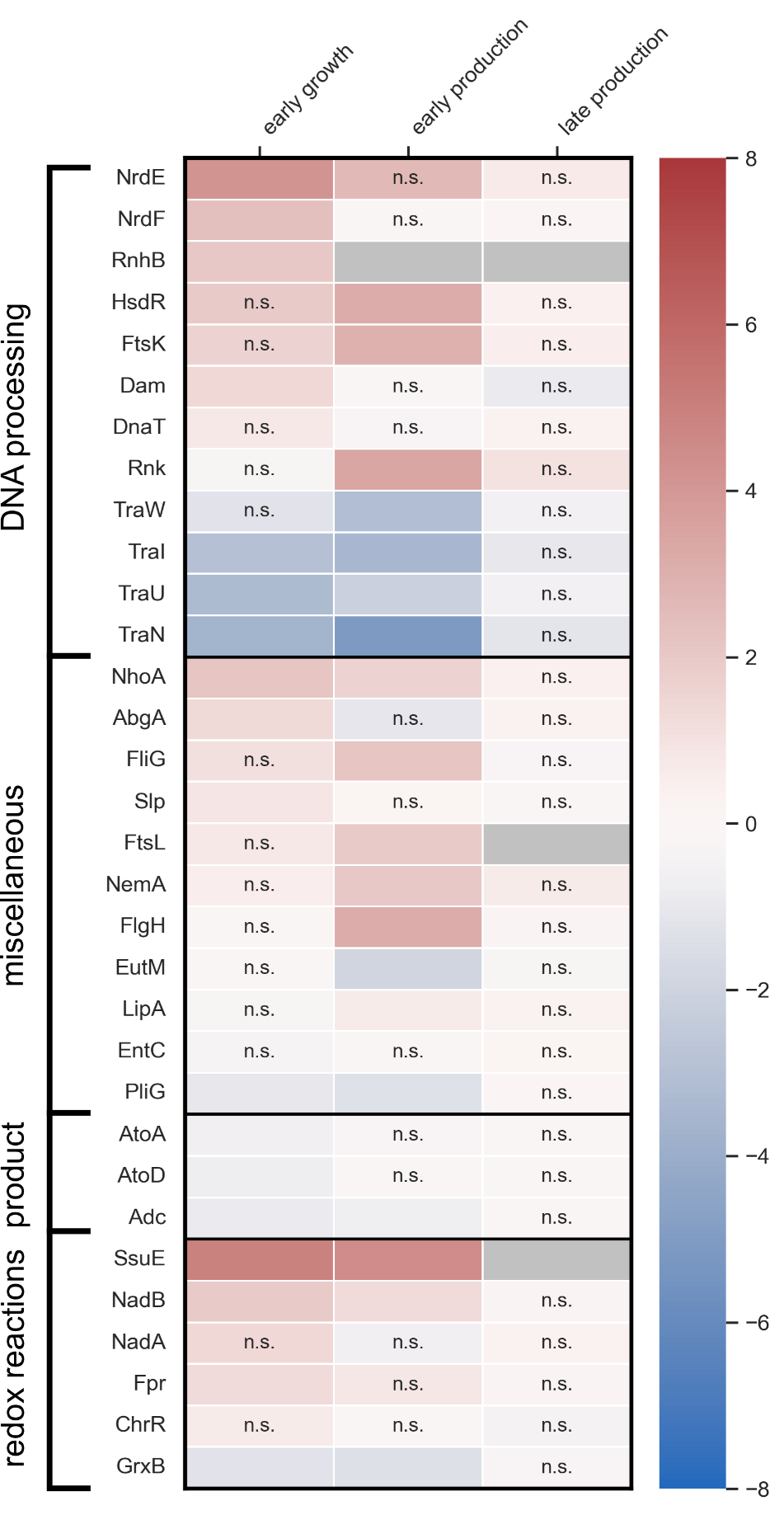


**Fig. S16.** Complete across-temperature log2 fold changes of proteins associated with DNA processing, redox reactions, product formation and miscellaneous. For every compared phase between the different temperature processes the 25 °C sample was used as a reference (therefore red and blue color indicates a higher abundance at 25 °C and 37 °C respectively). “n.s.” (“not significant”) indicates a non-significant fold change. Gray tiles denote that the protein was not detected in the sample.


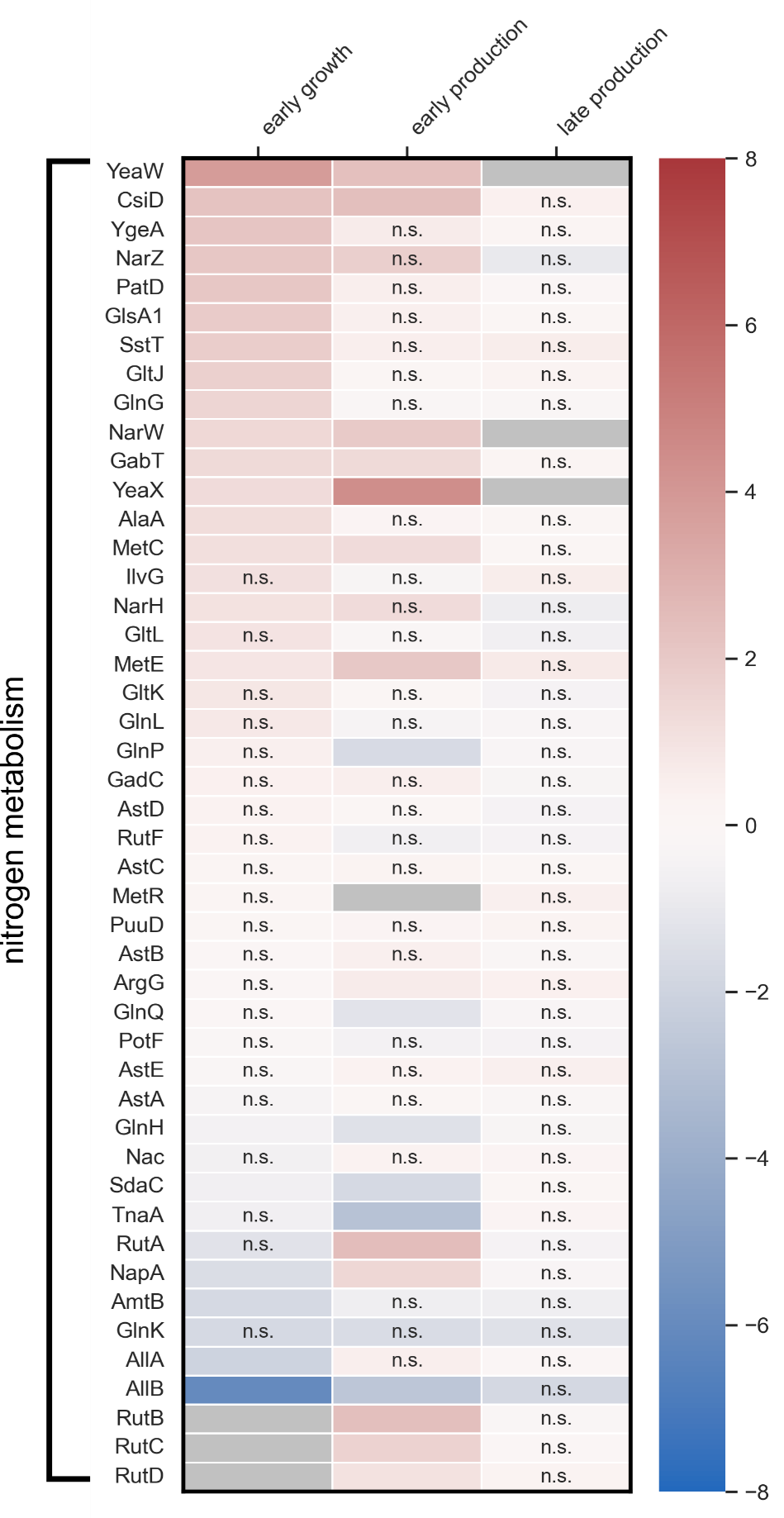


**Fig. S17.** Complete across-temperature log2 fold changes of proteins associated with nitrogen metabolism. For every compared phase between the different temperature processes the 25 °C sample was used as a reference (therefore red and blue color indicates a higher abundance at 25 °C and 37 °C respectively). “n.s.” (“not significant”) indicates a non-significant fold change. Gray tiles denote that the protein was not detected in the sample.


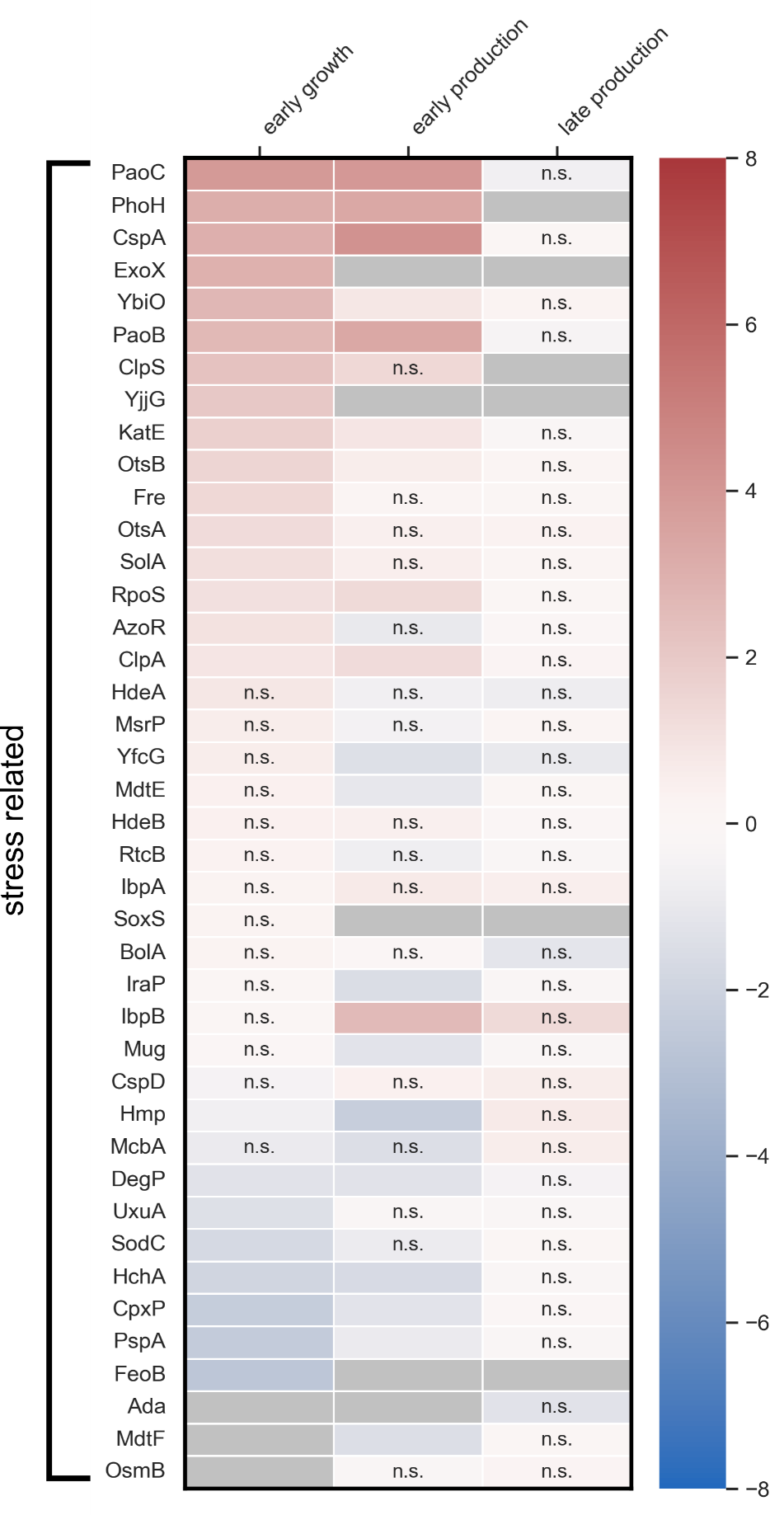


**Fig. S18.** Complete across-temperature log2 fold changes of proteins associated with stress. For every compared phase between the different temperature processes the 25 °C sample was used as a reference (therefore red and blue color indicates a higher abundance at 25 °C and 37 °C respectively). “n.s.” (“not significant”) indicates a non-significant fold change. Gray tiles denote that the protein was not detected in the sample.


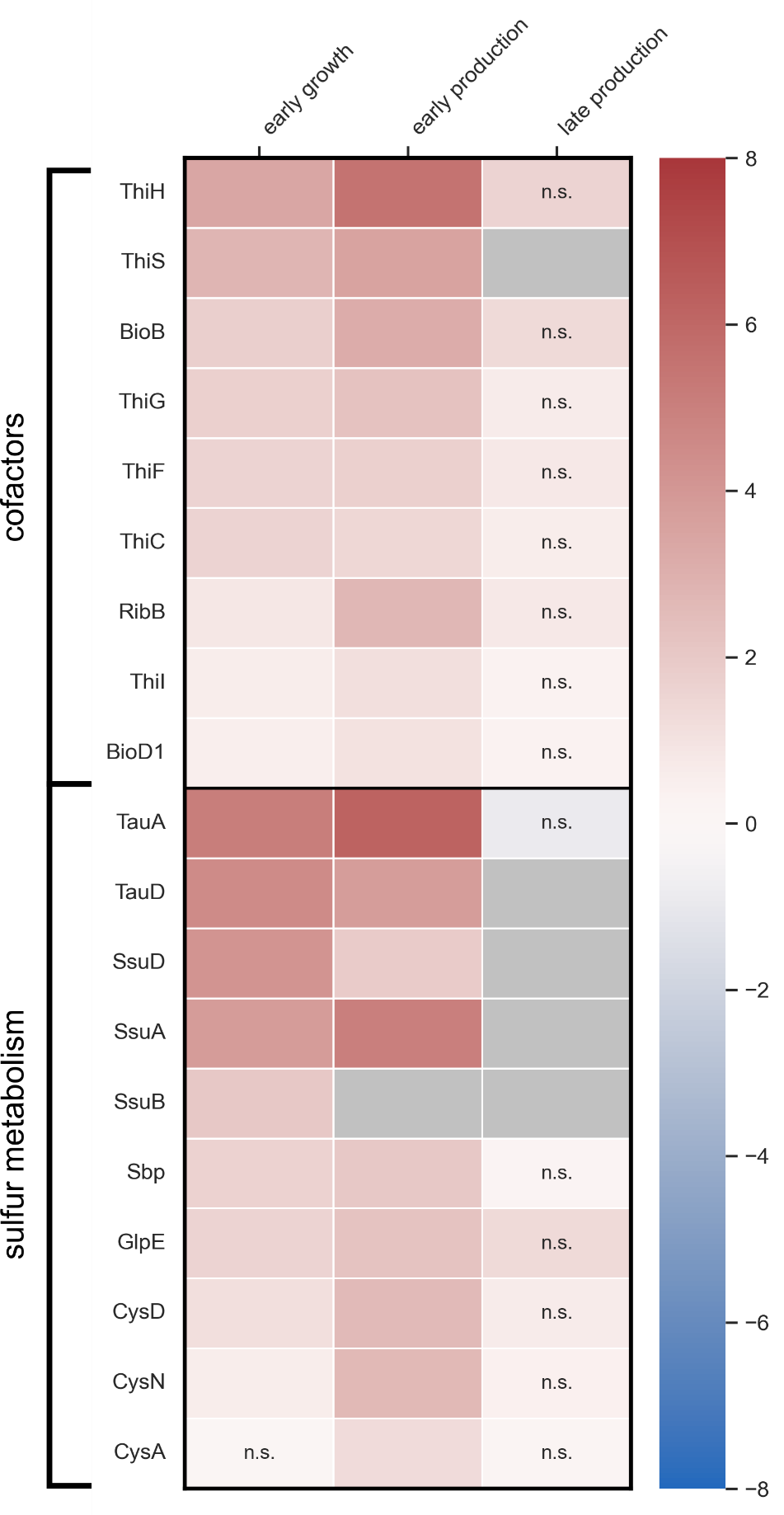


**Fig. S19.** Complete across-temperature log2 fold changes of proteins associated with cofactor formation and sulfur metabolism. For every compared phase between the different temperature processes the 25 °C sample was used as a reference (therefore red and blue color indicates a higher abundance at 25 °C and 37 °C respectively). “n.s.” (“not significant”) indicates a non-significant fold change. Gray tiles denote that the protein was not detected in the sample.


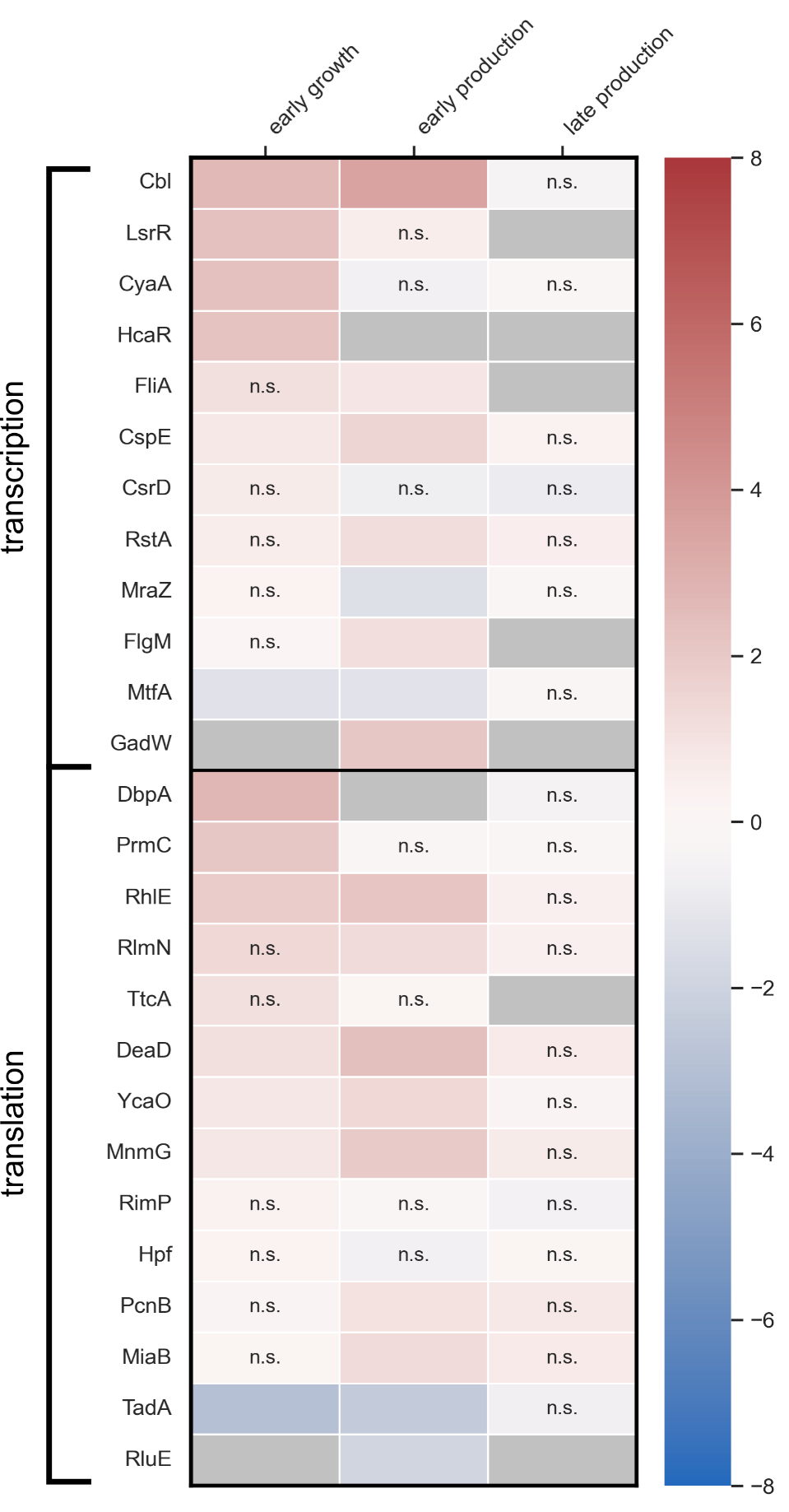


**Fig. S20.** Complete across-temperature log2 fold changes of proteins associated with transcription and translation. For every compared phase between the different temperature processes the 25 °C sample was used as a reference (therefore red and blue color indicates a higher abundance at 25 °C and 37 °C respectively). “n.s.” (“not significant”) indicates a non-significant fold change. Gray tiles denote that the protein was not detected in the sample.


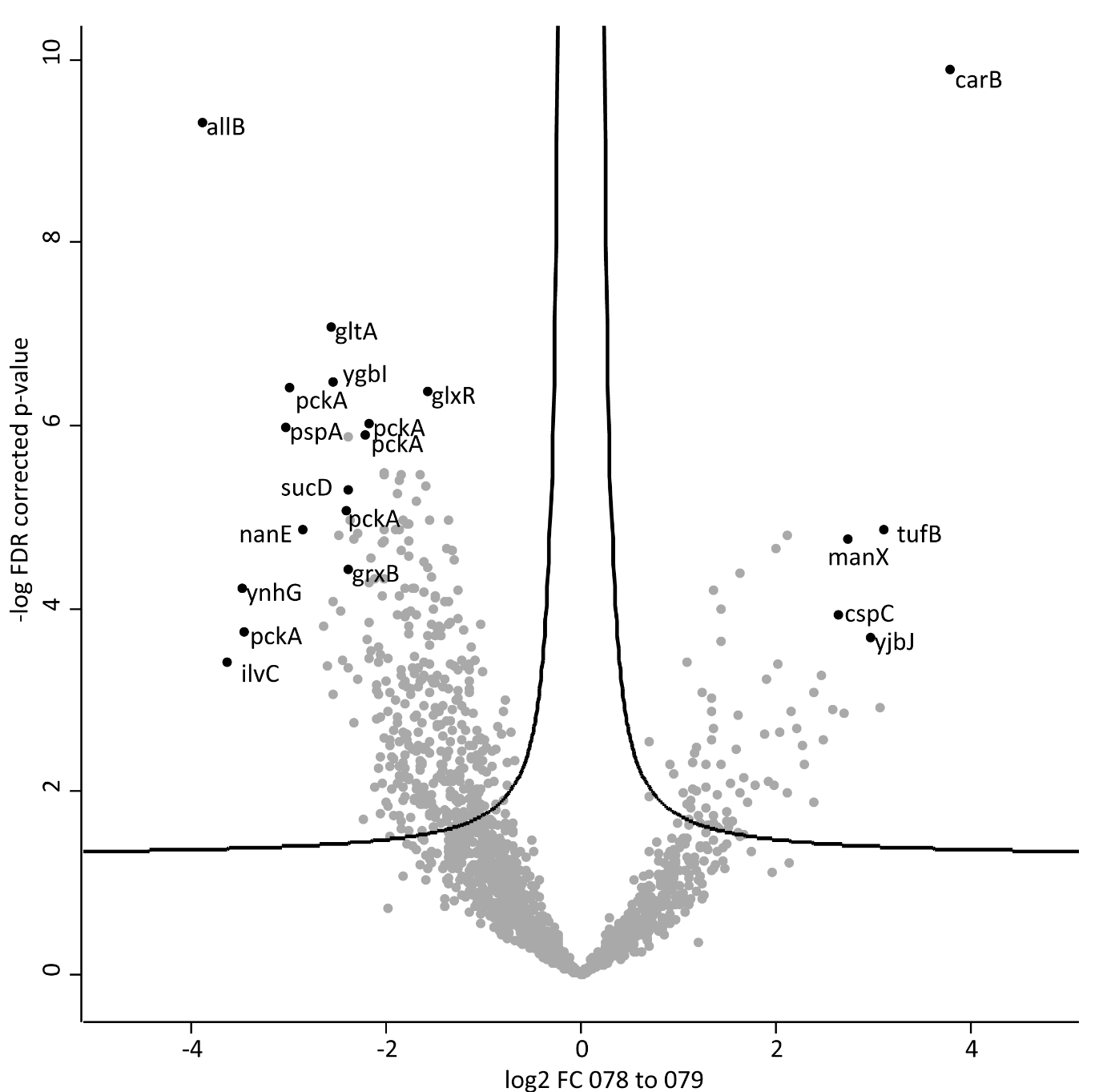


**Fig. S21.** Acetylated proteomics overall volcano plot.


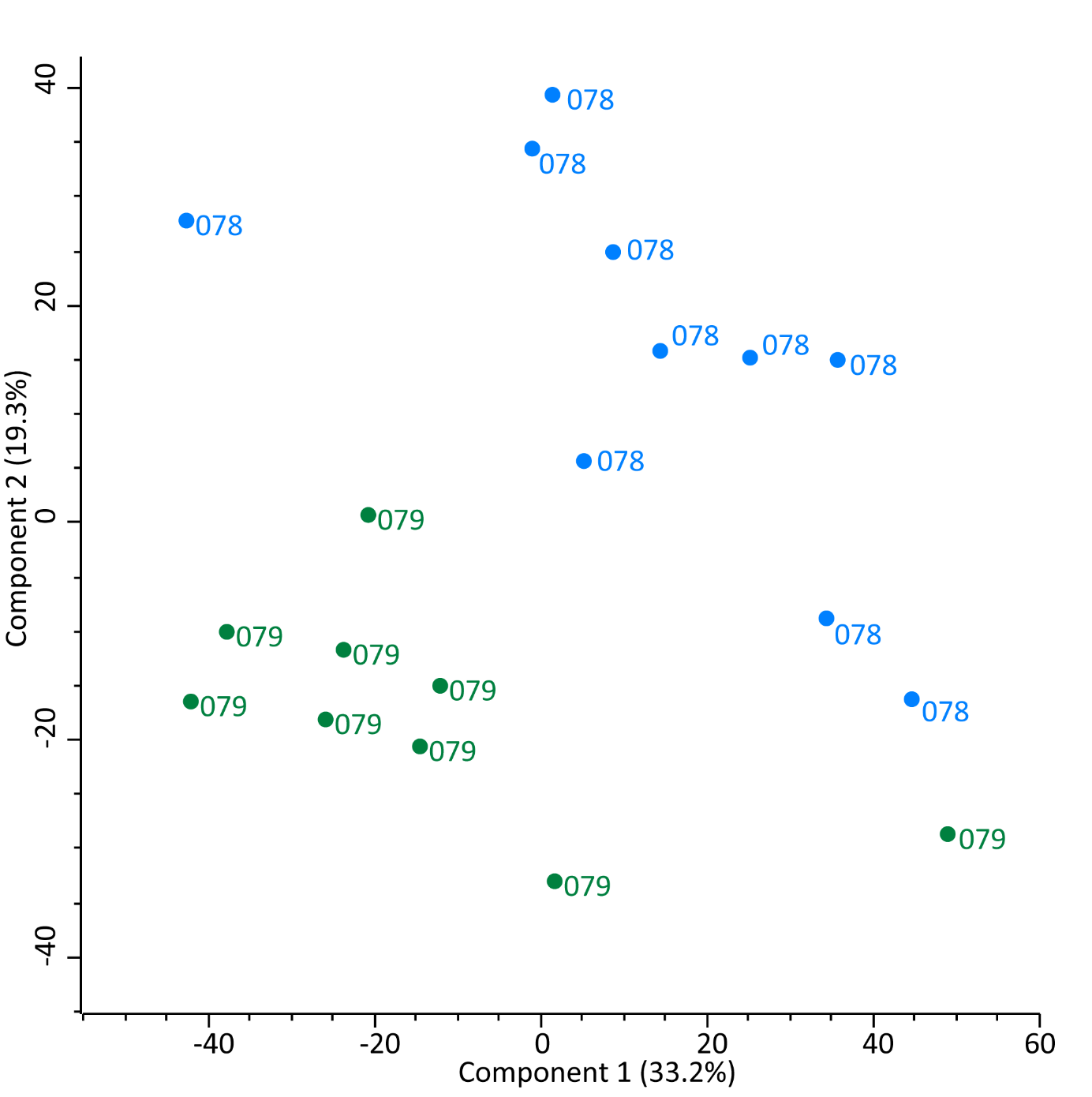


**Fig. S22.** Acetylated proteomics principal component analysis plot.


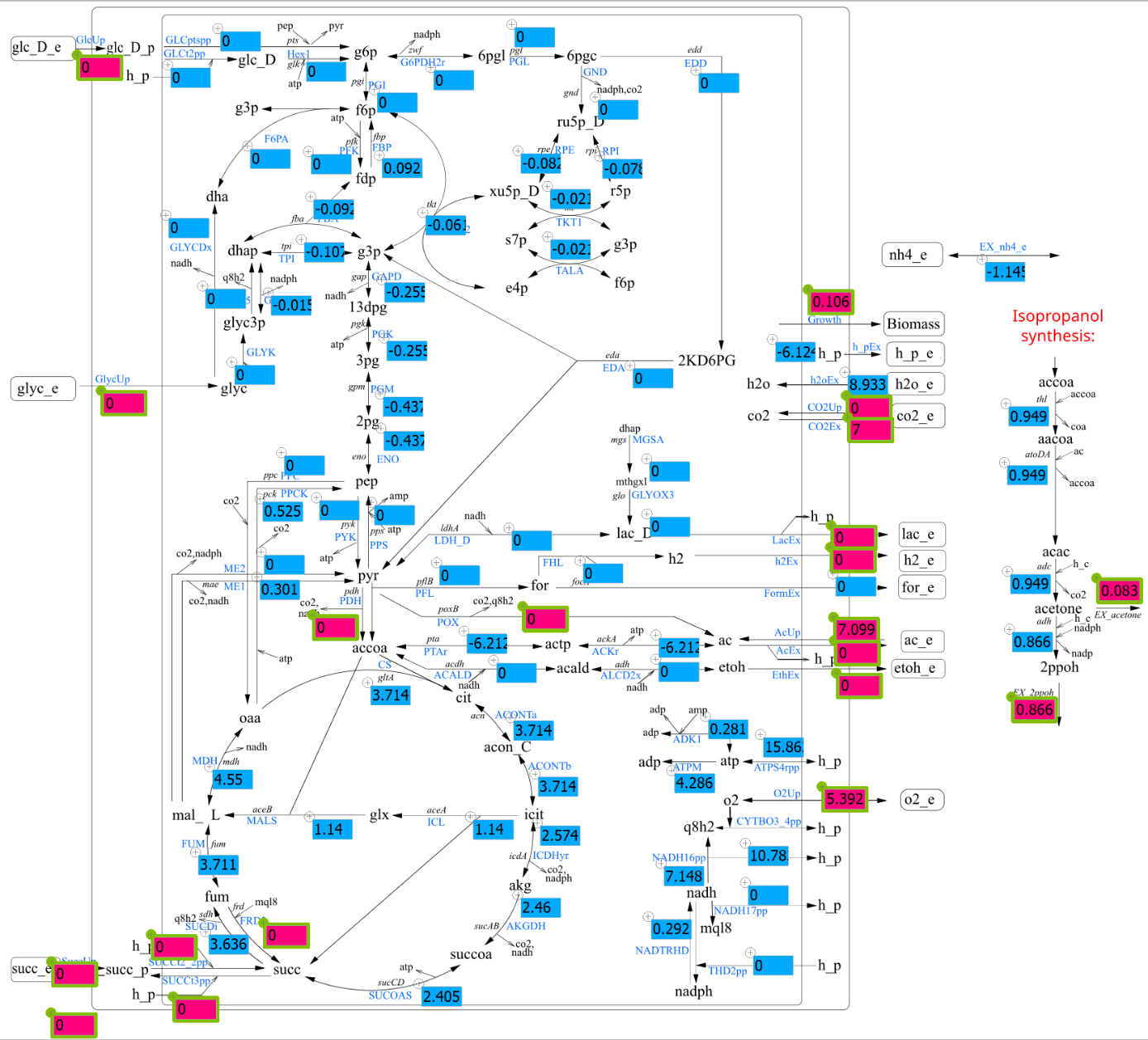


**Fig. S23.** Example of a flux scenario in the ECC2comp model in CNApy. In this case, sample 5 (33.9 h) of the 25 °C process on acetate is shown. Input (fixed) data is shown in red. Minor adjustments were performed by the model to the specific acetate uptake rate (original input value: 7.087 mmol g^‑1^ h^‑1^) and the specific oxygen uptake rate (original input value: 6.31 mmol g^‑1^ h^‑1^), as explained in the Methods. A full version of all input and model data is given in “Supplementary_file_2”.


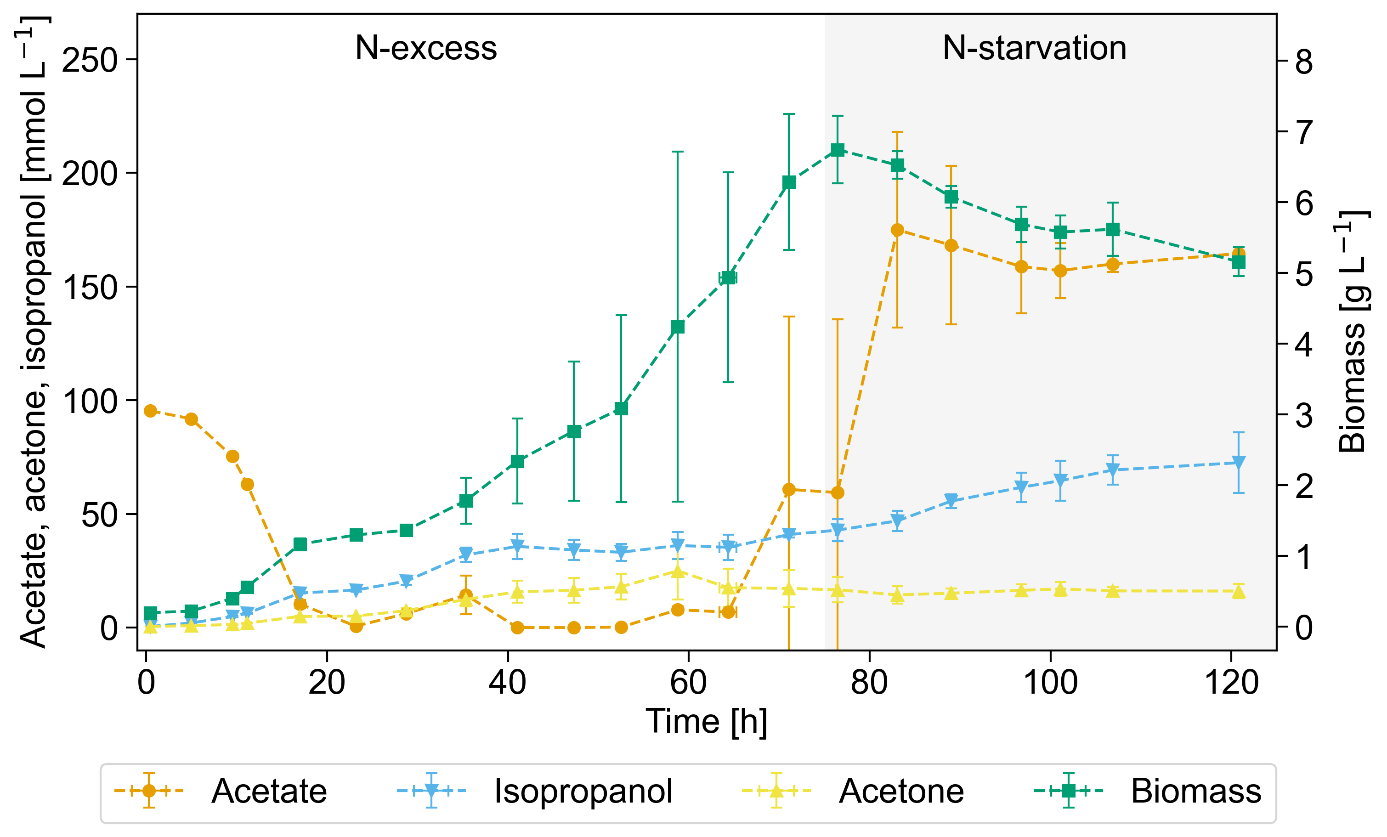


**Fig. S24.** Physiological data gained from cultivation of E. coli IPA_P_p105 at 25 °C implementing a carbon limitation during N-excess. Error bars show the standard deviation of 2 independent biological duplicates. The cells heavily favor biomass formation during the N-excess phase.


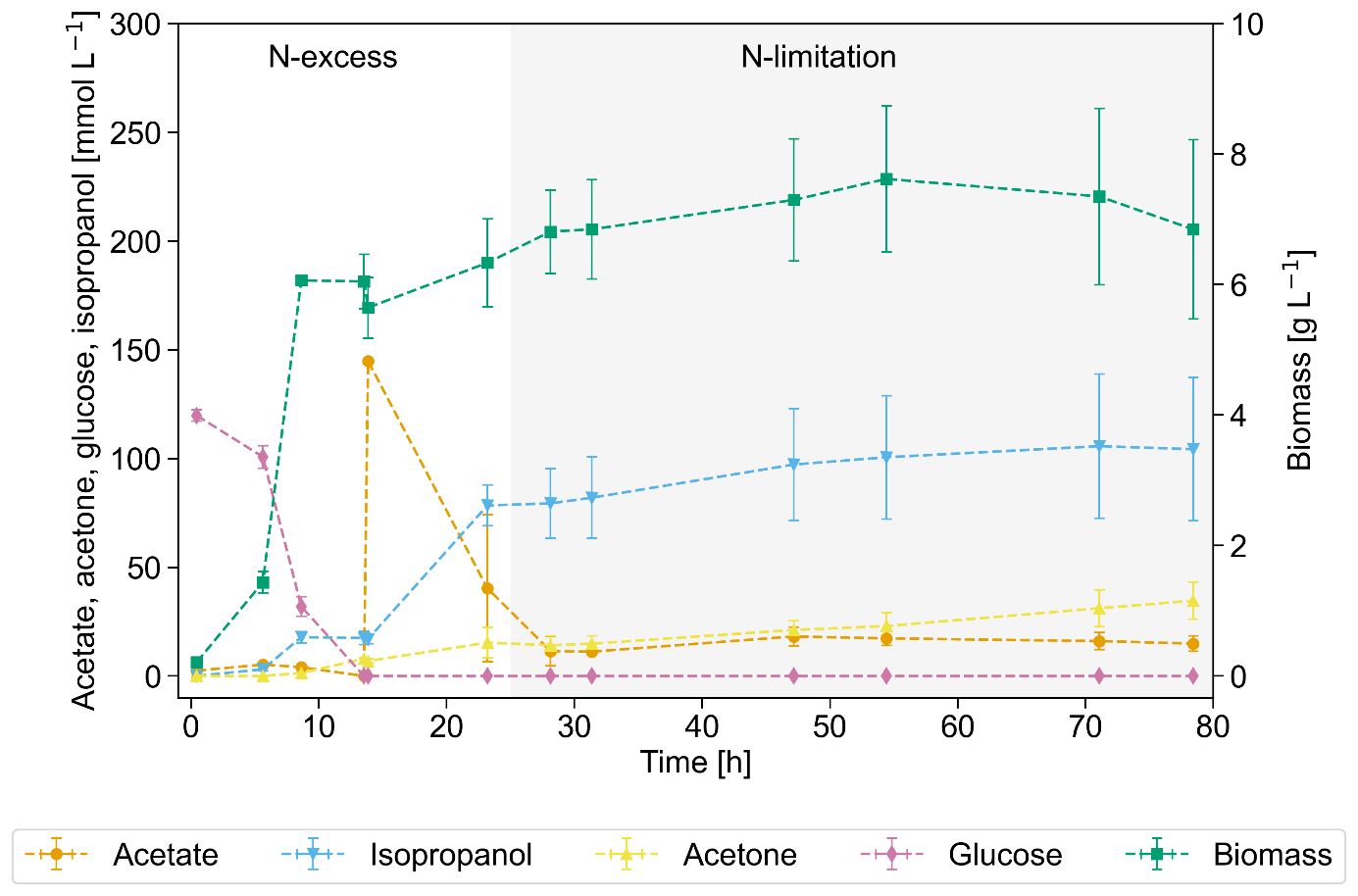


**Fig. S25.** Physiological data gained from cultivation of E. coli IPA_P_p105 at 25 °C implementing a nitrogen limiting feeding regime (target µ = 0.01 h^-1^). Error bars show the standard deviation of 2 independent biological duplicates. Glucose was used as initial carbon source to accelerate cell growth. The cells were then adapted to acetate, which was continuously provided during the N-limitation phase. While the N-limitation phase started with 6 – 7 g L^‑1^ of biomass, carbon was mainly used to generate more biomass instead of isopropanol.


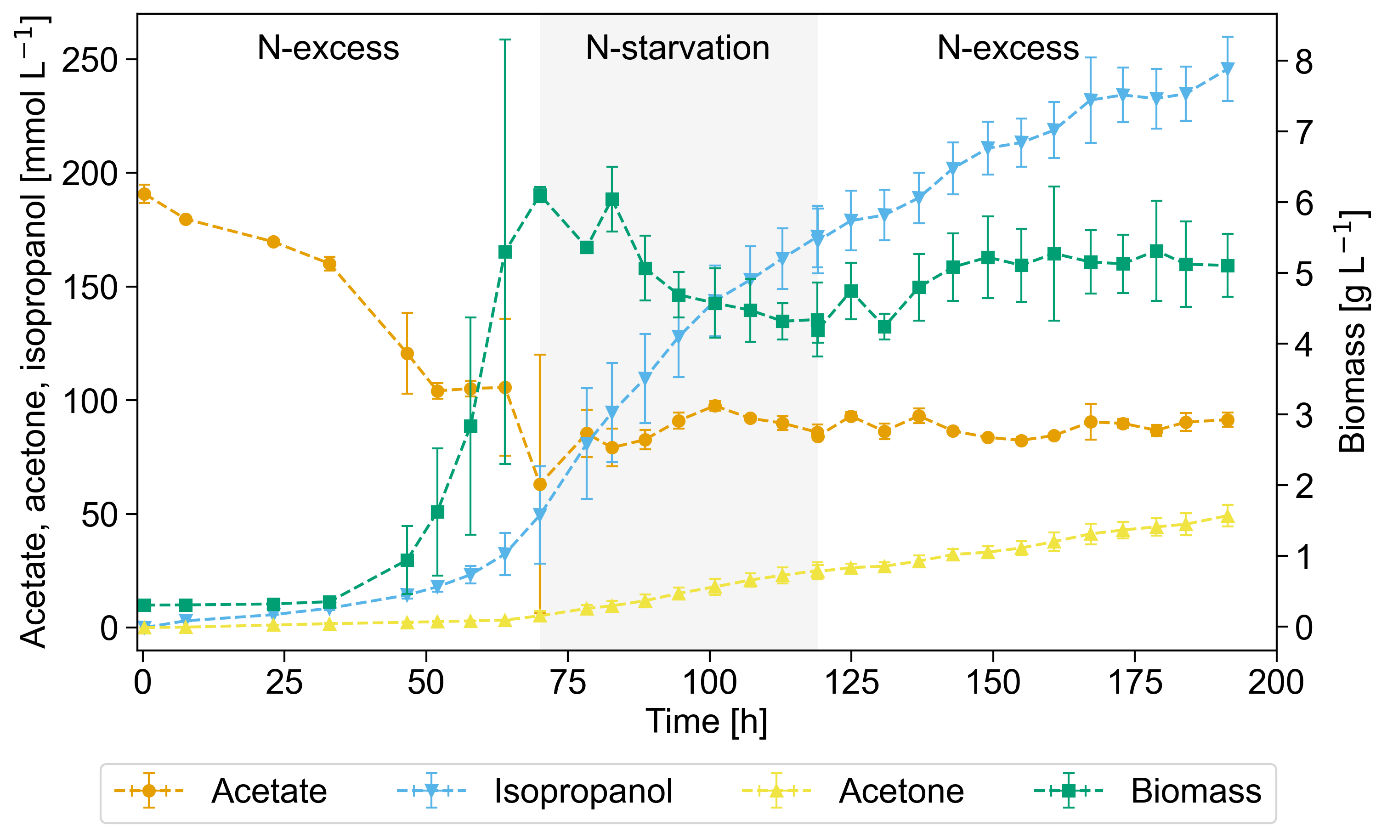


**Fig. S26.** Physiological data gained from cultivation of E. coli IPA_P_p105 at 25 °C implementing a 45-h nitrogen starvation followed by another N-excess phase. Error bars show the standard deviation of 2 independent biological duplicates. For the second N-excess phase, the cells were supplied with 20 mmol L^‑1^ of nitrogen ((NH_4_)_2_HPO_4_), but their nitrogen uptake was significantly lower than during the first N-excess phase, which resulted in only marginal cell growth (residual nitrogen at the end of the process: ~ 6 mmol L^‑1^). The continuous decline of the specific acetate uptake rate which started in the N-starvation phase could not be mitigated by adding more nitrogen.


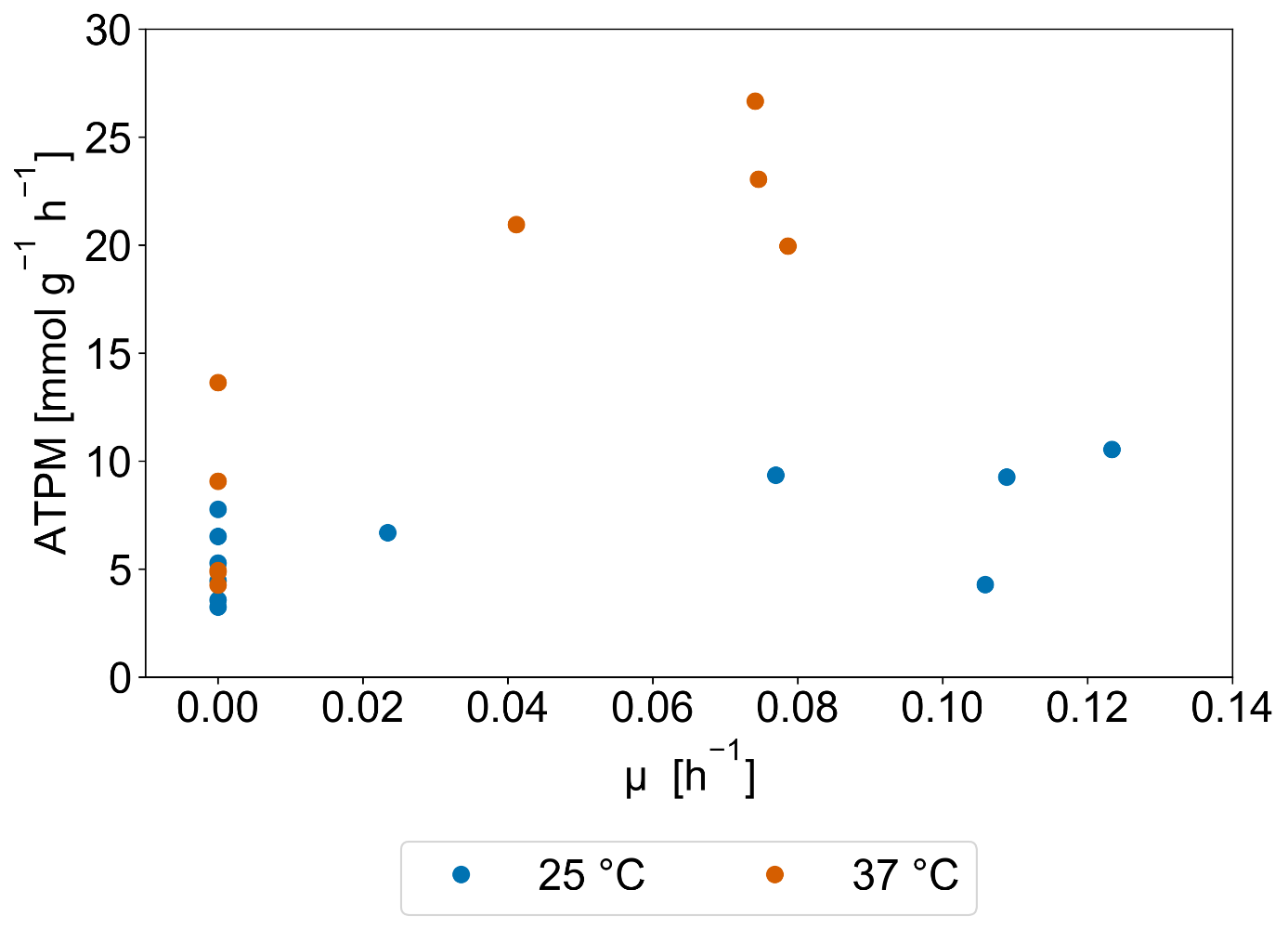


**Fig. S27**. Correlation between specific growth rate (µ) and non-growth-associated maintenance energy fluxes (ATPM) at different temperatures. A correlation between µ and ATPM indicates that the actual growth-dependent ATP demand is higher than assumed by for the metabolic model.

References

Sarkari, P., Marx, H., Blumhoff, M.L., Mattanovich, D., Sauer, M., Steiger, M.G., 2017. An efficient tool for metabolic pathway construction and gene integration for Aspergillus niger. Bioresource Technology, SI on Advances in Industrial Bioprocesses and Products- Genetic and Metabolic Engineering Interventions 245, 1327–1333. https://doi.org/10.1016/j.biortech.2017.05.004
